# A Multi‐Organ Atlas Links Gut Microbial Metabolites to Systemic Redox Changes in Aging Mice

**DOI:** 10.1111/acel.70433

**Published:** 2026-03-09

**Authors:** Sanaullah Sajid, Jieliang Huang, Shaofang Kong, Chengze Lai, Zhuoxin Tan, Yiming Shao, Lianxian Guo

**Affiliations:** ^1^ Dongguan Key Laboratory of Public Health Laboratory Science, the First Dongguan Affiliated Hospital, School of Public Health Guangdong Medical University Dongguan China; ^2^ Guangdong Provincial Key Laboratory of Medical Molecular Diagnostics, School of Medical Technology Guangdong Medical University Dongguan China; ^3^ Fogang County People's Hospital Qingyuan Guangdong China; ^4^ Dongguan Key Laboratory of Sepsis Translational Medicine, the First Dongguan Affiliated Hospital Guangdong Medical University Dongguan China

**Keywords:** ferroptosis susceptibility, gut microbiota, meta‐analysis, multi‐omics, oxidative stress, systemic inflammaging

## Abstract

Aging disrupts systemic metabolism, but the mechanisms by which gut microbial metabolites drive tissue‐specific decline remain unclear. We conducted a multi‐organ, multi‐omics atlas across the gut, serum, liver, lung, and cortex in young and early‐aged mice to address this. We identified a conserved aging signature marked by the microbiota‐associated depletion of protective circulating metabolites, such as lysophosphatidylcholines (LPCs), concurrently with the systemic accumulation of pro‐oxidative microbial catabolites, specifically trimethylamine N‐oxide (TMAO) and indole‐3‐acetic acid (IAA). This microbial‐metabolic drift disrupted systemic lipid transport and redox balance, leading to distinct organ‐level vulnerabilities: hepatic lipid retention and ferroptosis susceptibility, pulmonary immune‐redox activation, and cortical neurochemical dysregulation. To establish functional relevance, we conducted an integrated meta‐analysis of 40 independent studies encompassing natural aging models, fecal microbiota transplantation (FMT), and probiotic interventions. This quantitative synthesis provided convergent evidence that microbial remodeling is a functionally relevant correlate associated with systemic aging phenotypes by restoring intestinal barrier integrity (upregulating ZO‐1, MUC2), suppressing tissue inflammatory factors (IL‐6, IL‐1β, TNF‐α), and mitigating oxidative stress (reducing MDA and restoring SOD/GSH). Together, our findings highlight gut‐derived metabolic reprogramming as a modifiable, upstream driver of systemic aging, offering tractable targets for therapeutic intervention.

## Graphical abstract

A gut‐derived metabolic signature drives systemic aging phenotypes. Integrated multi‐omics profiling of young versus aged mice identifies a conserved aging signature characterized by the depletion of protective lysophosphatidylcholines (LPC 22:0) and the concurrent accumulation of pro‐oxidative microbial catabolites (TMAO, IAA). This metabolic drift propagates across the serum‐liver‐lung‐cortex axis, driving systemic redox stress and organ‐specific decline. A comprehensive meta‐analysis of 50 independent studies provides translational validation, demonstrating that reversing this signature via microbiome interventions restores intestinal barrier integrity (ZO‐1), suppresses inflammation (IL‐6), and boosts antioxidant capacity (SOD/GPX), thereby establishing the gut‐metabolite axis as a modifiable redox hub for extending healthspan.

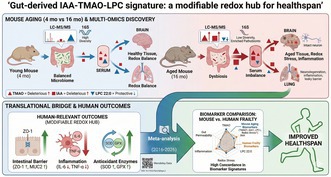

## 1 Introduction

Aging is a universal process characterized by progressive functional decline and increased vulnerability to chronic disease across all organ systems. Hallmark molecular changes, including mitochondrial dysfunction, oxidative stress, and chronic inflammation, have been well documented. However, the organization of these processes across different organs remains poorly understood (Franceschi et al. 2007; Hamidu et al. 2025). The gut has emerged as a central source of biochemical signals, generating metabolites and lipids that circulate systemically and influence distant tissues. Despite these observations, the coordinated system‐wise propagation of gut‐derived factors in driving multiple, specific tissue‐aging trajectories remains unclear (Wen et al. 2024). Previous work has primarily focused on single organs or biofluids, lacking the integrated framework necessary to define the systemic handoff of stress. Our work uniquely provided the first multi‐organ, multi‐omics atlas linking the gut‐serum‐liver‐lung‐cortex axis, defining conserved and tissue‐specific vulnerabilities in the systemic context previously missing from multi‐tissue studies.

The gut microbiome has emerged as a key regulator of host physiology during aging. Age‐associated dysbiosis alters the production of short‐chain fatty acids, bile acids, indole derivatives and metabolites that modulate systemic redox balance, immune function, and mitochondrial activity (Jing et al. 2025; Zhang, Li, et al. 2025). Observational studies in humans have linked microbial shifts with frailty, cognitive decline, and chronic inflammation. Furthermore, microbiota transplantation experiments demonstrated that aged microbiomes can accelerate physiological decline when transferred into young hosts (Jing et al. 2025; Olejnik et al. 2025). Together, these findings suggested that microbial remodeling may act as an upstream organizational driver of systemic metabolic drift rather than simply accompany aging (Ghosh et al. 2022).

Metabolomics studies have provided important insights into these processes but have largely focused on individual tissues or biofluids. Analyses of serum or brain, for example, revealed age‐related signatures of lipid peroxidation, antioxidant depletion, and neurotransmitter imbalance. Yet such single‐compartment approaches cannot capture the coordinated metabolic exchanges that occur across organs (Jové et al. 2014). For instance, a study (Yiming et al. 2023) profiled human serum and identified 349 metabolites (including lysophospholipids) associated with age, while another study (Ding et al. 2021) generated a metabolome atlas of the aging mouse brain across regions. These single‐organ studies highlighted important changes, but Aging is inherently systemic, with organs displaying distinct yet interconnected vulnerabilities (Gao et al. 2022). A comprehensive strategy is therefore required to define conserved molecular signatures and organ‐specific adaptations that collectively shape aging trajectories (Ahadi et al. 2020).

Here, we address this gap by performing pseudo‐targeted metabolomics across the gut, serum, liver, lung, and cortex in mice spanning young (4 months) to early‐aged (16 months). This age point was specifically selected to capture the early metabolic remodeling that precedes the onset of severe frailty and irreversible tissue damage. By integrating these multi‐organ profiles with gut microbiome sequencing, we aimed to: (1) delineate how microbial remodeling reshapes circulating metabolite pools and contributes to tissue‐specific aging processes, specifically by driving systemic redox changes. Here, we define redox changes as the loss of endogenous antioxidant capacity (e.g., glutathione and NAD^+^) and the concurrent accumulation of lipid peroxidation products (e.g., MDA and 13‐HODE) that collectively lower the cellular threshold for ferroptosis‐like remodeling (Covarrubias et al. 2021; Jiang et al. 2021); (2) identify conserved mechanistic pathways, including the tryptophan‐indole‐kynurenine pathway and LPC‐PUFA remodeling pathway, as potential systemic correlates of metabolic aging and characterize their multi‐organ convergence on ferroptosis susceptibility; and (3) nominate tractable microbial and lipid mediators as biomarkers and potential intervention targets. This multi‐organ, multi‐omics atlas provides a detailed mechanistic framework for mid‐life metabolic remodeling, identifying how coordinated but tissue specific pre‐aging signatures develop across the system.

## 2 Methods

### 2.1 Ethical Compliance

All animal procedures were approved by the Institutional Animal Care and Use Committee of Guangdong Medical University (Approval No. GDY2004001) and were conducted in accordance with national and institutional guidelines for the ethical treatment of laboratory animals. Study protocols followed the 3Rs principles of Replacement, Reduction, and Refinement to minimize animal use and suffering while maintaining statistical rigor.

### 2.2 Animal Cohorts and Study Design

Mice were housed in a specific‐pathogen‐free (SPF) facility with controlled environmental conditions: 12‐h light/dark cycle, temperature maintained at 22°C ± 1°C, and relative humidity at 55% ± 10%. Animals were kept in individually ventilated cages with ad libitum access to standard laboratory chow and sterilized water (Sajid et al. 2025). To isolate physiological aging effects, we established a cross‐sectional, multi‐organ profiling design using two age groups of male C57BL/6 mice (initial *n* = 10 per group). The young adult group (CM4) consisted of 4‐month‐old mice, representing early adult physiology, while the aged group (CM16) included 16‐month‐old mice, a time point reflecting early aged in murine lifespan and corresponding to mid‐life metabolic changes in humans (approx. 45–50 human years). These animals were derived from the same experimental batch as a parallel study, where they served as the control cohort for an investigation into long‐term environmental exposure (Lai et al. 2024). Importantly, no pharmacological, dietary or environmental interventions were applied to these animals between 4 and 16 months. This ensured we specifically studied the endogenous trajectory of natural metabolic aging under controlled SPF conditions. Mice were monitored for health status, and aged animals did not show signs of illness beyond normal age‐related phenotypes. All downstream sample collection, LC–MS acquisition, and data analyses were conducted in a fully randomized and blinded manner (Cheng et al. 2022; Lai et al. 2024).

### 2.3 Tissue Collection and Biospecimen Processing

At the designated ages (4 or 16 months), mice were anesthetized and euthanized for tissue harvest. Tissues were rapidly dissected, flash‐frozen in liquid nitrogen, and stored at −80°C until analysis. We harvested five biological matrices from each animal: (1) fecal pellets for profiling of microbiota and derived metabolites, (2) whole blood for serum separation as the systemic circulation conduit, (3) liver as the principal site of host‐microbial metabolic interface and detoxification, (4) lung as an underexplored peripheral organ sensitive to redox stress and microbial infiltration, and (5) cerebral cortex to examine neurochemical aging and blood–brain barrier integrity. Tissue handling was conducted in a fully randomized and blinded fashion to prevent technical bias. All samples were processed within 30 min of collection to ensure preservation of labile redox‐sensitive metabolites and lipids (Lai et al. 2024).

### 2.4 Pseudo‐Targeted Metabolomic Profiling

Pseudo‐targeted metabolomic profiling for all tissues was performed using high‐performance liquid chromatography coupled with tandem mass spectrometry (HPLC‐MS/MS), strictly adhering to the protocols established in our previous research. Portions of the raw data utilized from our previously published research on arsenic exposure studies (Lai et al. 2024; Sajid et al. 2025). Analytes were detected in Multiple Reaction Monitoring (MRM) mode based on a priori defined ion pairs (precursor‐to‐product transitions) and retention times. Raw MS data were processed for peak integration and intensity extraction. Identified features were imported into R 4.0.2 for downstream processing. Missing values were imputed using the Random Forest algorithm, and metabolite intensities were Log2 transformed to ensure normality. Differential analysis was conducted using Orthogonal Partial Least Squares Discriminant Analysis (OPLS‐DA) to identify features distinguishing aged from young groups, with significance criteria set at VIP > 1 and *p* < 0.05 (Student's *t*‐test). Pathway enrichment analysis for differentially expressed metabolites was performed using MBROLE 2.0 (https://csbg.cnb.csic.es/mbrole2), with visualizations generated via the R‐circlize package (Lai et al. 2024).

### 2.5 Assessment of Redox, Ferroptosis, and Barrier Markers via Pseudo‐Targeted Profiling

To investigate ferroptosis‐like metabolic remodeling, we analyzed the relative abundance of characteristic markers detected across tissues via pseudo‐targeted HPLC‐MS/MS. Relative levels of reduced (GSH) and oxidized glutathione (GSSG) were assessed based on normalized peak intensities to infer systemic redox status. Similarly, lipid peroxidation markers, including Malondialdehyde (MDA) and 13‐hydroxyoctadecadienoic acid (13‐HODE), were monitored to identify features distinguishing aged from young tissues. Long‐chain acylcarnitines and polyunsaturated fatty acids (PUFAs) were evaluated to characterize patterns associated with mitochondrial dysfunction and ferroptotic lipid accumulation. Lysophosphatidylcholines (LPCs) were assessed based on relative ion intensities to determine systemic changes (Tang et al. 2021). We also tracked the relative signal intensities of neuroactive metabolites (e.g., dopamine, glutamate, GABA) and purine intermediates (e.g., guanosine, xanthine) in the cortex to infer shifts in neurotransmitter balance. Notably, the detection of normally gut‐restricted metabolites such as taurocholate and indole‐3‐acetic acid (IAA) in the cortex, supported by multi‐organ correlation networks, was utilized as inferential evidence of age‐associated blood–brain barrier compromise. These pseudo‐targeted profiles collectively enabled a mechanistic interpretation of systemic aging phenotypes based on comparative fold‐changes and pathway enrichment rather than absolute quantification.

### 2.6 Microbiome Analysis (16 s rRNA Sequencing and Functional Prediction)

Microbial community profiling and functional prediction were performed strictly adhering to the protocols established in our previous research (Lai et al. 2024; Sajid et al. 2025). Microbial community composition was assessed by 16S rRNA gene sequencing of fecal samples. DNA was extracted, and the V4‐V5 hypervariable region of the 16S gene was amplified and sequenced on an Illumina MiSeq platform (2 × 250 bp). Reads were quality‐filtered, denoised, and chimera‐removed in QIIME2 (v2023.7) using the DADA2 pipeline to generate amplicon sequence variants (ASVs). Taxonomic assignment was performed against the SILVA 138 database at 97% identity. Alpha diversity was calculated using Shannon, Simpson, dominance and Chao1 indices. Beta‐dispersion homogeneity was evaluated using PERMDISP prior to group testing. PERMANOVA was performed on Bray‐Curtis distances. LEfSe was run with *α* = 0.05, LDA > 2.0, and multi‐test correction applied at the Kruskal–Wallis stage to control false discoveries. To infer microbial function, metagenome predictions were generated using PICRUSt2, producing KEGG orthologs (KOs) and MetaCyc pathway profiles. The reliability of predictions was assessed with the nearest sequenced taxon index (NSTI), which averaged < 0.1 across samples, indicating strong functional inference. Group differences in KO and pathway abundance were evaluated by Wilcoxon tests with Benjamini‐Hochberg false discovery rate correction (Hong et al. 2024; Ma et al. 2024).

### 2.7 Causality Validation: Systematic Review and Meta‐Analysis

To address potential causality concerns regarding the correlational nature of our cross‐sectional findings, we conducted a comprehensive systematic literature review and meta‐analysis following PRISMA guidelines (Page et al. 2021). Our search strategy focused on identifying studies that evaluated gut microbiota alterations, fecal microbiota transplantation (FMT), and probiotic interventions in the context of aging. We systematically searched PubMed and Google Scholar for literature published between 2016 and January 2026. The search utilized the following keyword combinations: ((“gut microbiota” OR “microbiome” OR “fecal microbiota transplantation”) AND (“aging” OR “longevity” OR “senescence”)) AND (“inflammation” OR “C‐reactive protein” OR “interleukin‐6” OR “IL‐6” OR “TNF‐alpha” OR “ZO‐1” OR “occludin” OR “claudin”) AND (“SOD” OR “MDA” OR “GSH”).

Studies were strictly included if they met the following criteria: (1) published between 2016 and January 2026; (2) established mouse models of natural aging, evaluated the transplantation of fecal microbiota from young mouse donors to aged mice, or assessed probiotic supplementation in aged mice; (3) reported quantitative outcomes for specific inflammatory factors (IL‐10, IL‐6, IL‐1β, TNF‐α), barrier indicators (ZO‐1, Cldn1, Ocln1, MUC2), oxidative stress indicators (MDA, SOD, GSH), or other key systemic markers including I‐FABP, Endotoxin, PAI‐1, p16, and MCP‐1; and (4) clarified the specific tissue or detection area for these indicators. Quality assessment and statistical integration (including random‐effects modeling and heterogeneity evaluation) were conducted to synthesize the standardized mean differences across these distinct intervention strategies.

### 2.8 Statistical and Multi‐Omics Integration

Metabolite differences between CM4 and CM16 were tested using two‐tailed Student's *t*‐tests with Welch's correction. For univariate comparisons, *p*‐values were adjusted by Benjamini‐Hochberg FDR independently within each matrix and tissue combination to maintain rigor across the five distinct omics datasets. We report effect sizes (Hedges' g) with 95% CIs. Volcano plots use uniform thresholds of **|**log2 fold‐change| ≥ 1 (≥ 2.0×) and *q* < 0.05 across all tissues to standardize interpretation. Multivariate analyses included principal component analysis (PCA) and hierarchical clustering of Z‐score normalized data. Pathway enrichment was conducted using MetaboAnalyst 5.0 with KEGG pathway libraries, applying Fisher's exact test and topological impact scoring. Integration of microbiome and metabolome data was performed by calculating Spearman correlations between key microbial taxa or pathways and serum or tissue metabolites. To account for the high number of comparisons, all correlation *p*‐values were adjusted using the Benjamini‐Hochberg False Discovery Rate (FDR) correction. Only associations with an adjusted *p* (*q*‐value) < 0.05 were considered statistically significant and included in the correlation networks and matrices. Inter‐organ relationships were visualized through chord diagrams, heatmaps, and radar plots. All analyses were performed in R (v4.1.2) and Python (v3.9), and figures were generated with GraphPad Prism (version 9.5.1), as well as in‐house scripts to create the specialized multi‐omics diagrams (Sajid et al. 2025). To ensure the validity of our multi‐organ integration, all biological matrices (feces, serum, liver, lung, and cortex) were synchronously harvested from the same individual animals. All procedures, from sample collection to LC–MS acquisition and data analysis, were conducted in a fully randomized and blinded manner to minimize technical bias. Sample sizes (*n* = 9 for CM4; *n* = 8 for CM16) were determined to be sufficient to detect significant metabolic shifts based on expected effect sizes, a premise subsequently supported by the consistent directional changes observed in our integrated meta‐analysis of natural aging models (Li et al. 2024).

## 3 Results

### 3.1 Gut Microbiota Diversity and Composition During Natural Aging

**FIGURE 1:**
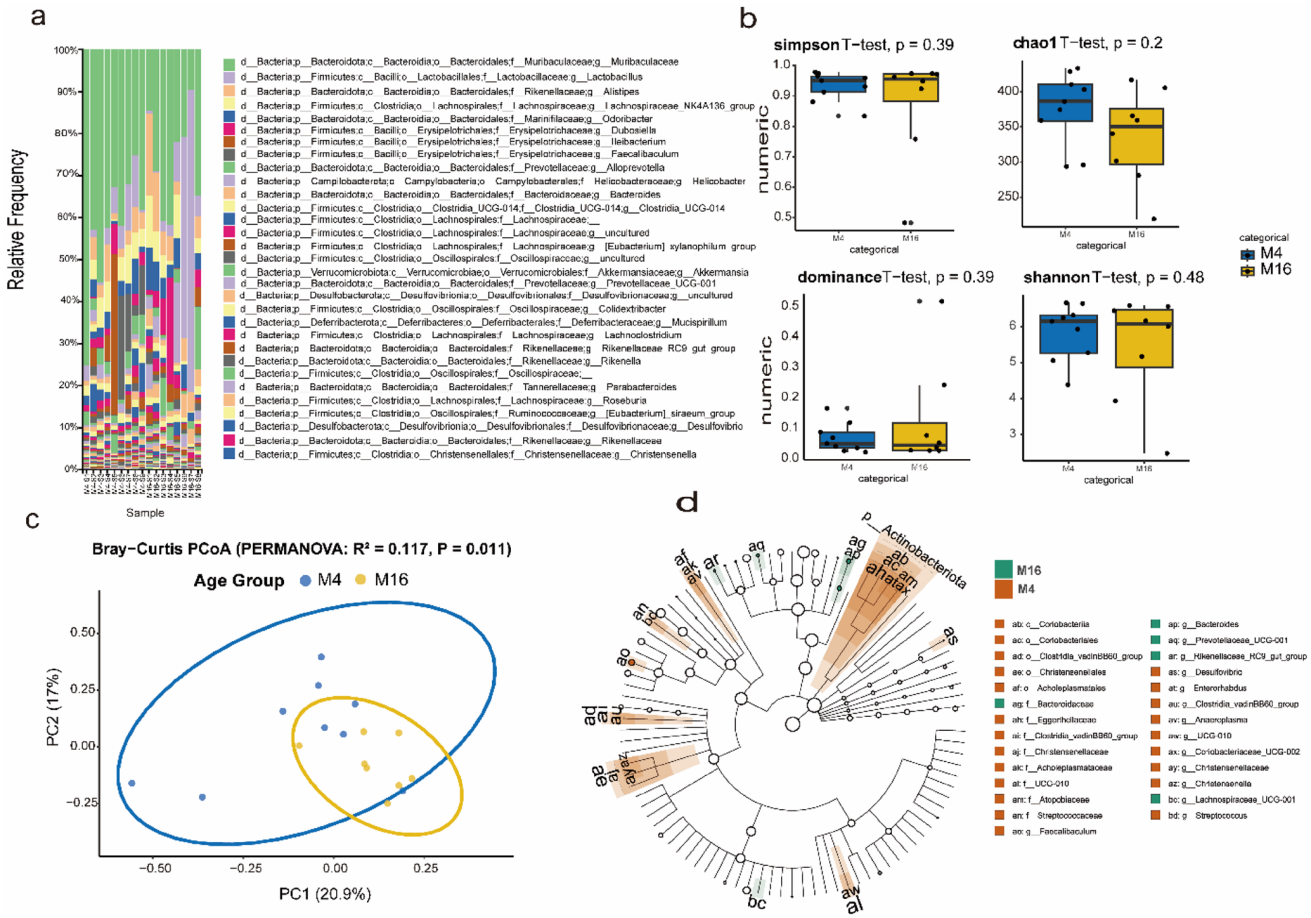
Gut microbiota compositional and functional remodeling marks the onset of aging. (a) Relative abundance of bacterial taxa across individual samples from young and aged mice, showing compositional restructuring with age. (b) Boxplots illustrating α‐diversity indices (Simpson, Chao1, Dominance, Shannon) revealed no significant differences between groups (all *p* > 0.2, Student's *t*‐test), indicating diversity is preserved. (c) Principal coordinate analysis (PCoA) based on Bray‐Curtis dissimilarities demonstrated clear separation of microbial communities between CM4 and CM16 (PERMANOVA, *R* ^*2*^ = 0.117, *p* = 0.011). (d) LEfSe cladogram identified age‐discriminant taxa, with *Bacteroides* and *Rikenellaceae* enriched in CM16 (Aged), and *Christensenellaceae*, *Coriobacteriaceae*, *Faecalibaculum*, and *Actinobacteria* enriched in CM4 (Young). Together, these analyses highlight a structured reorganization of the aged microbiota characterized by compositional shift, but not diversity loss.

We analyzed gut microbiota profiles from young (4‐month‐old, CM4) and aged (16‐month‐old, CM16) mice after applying quality control filters (excluding samples with insufficient sequencing depth or missing metadata). The resulting community profiles enabled us to test whether natural aging influences microbial diversity and community structure (Figure 1a). Alpha‐diversity analysis showed that overall richness and evenness of the gut microbiome were largely preserved with aging. Shannon, Simpson, Chao1, and Dominance indices did not differ significantly between CM4 and CM16 (all *p* > 0.20; Figure 1b). These results indicated that natural aging in mice does not reduce the total diversity of the gut microbial community. In contrast, beta‐diversity analysis revealed a clear restructuring of microbial composition with age. Bray‐Curtis dissimilarities demonstrated strong separation of CM4 and CM16 communities in principal coordinate space (PERMANOVA, *R* ^*2*^ = 0.117, 999 permutations, *p* = 0.011; Figure 1c). This compositional divergence was consistently observed across alternative distance metrics, confirming that aging is associated with a shift in microbial community structure beyond stochastic variation.

Taxonomic profiling provided insight into the lineages driving this divergence. At the family level, aged mice (CM16) exhibited significant enrichment of *Rikenellaceae* and *Bacteroidaceae*, while young mice (CM4) were characterized by higher abundance of *Christensenellaceae* and *Coriobacteriaceae* (Figure 1d). At the genus level, aged mice were enriched for *Bacteroides*, whereas young mice had higher levels of *Faecalibaculum*. These compositional patterns suggested that natural aging promotes the expansion of specific *bacteroidal* lineages. To validate these observations, we applied LEfSe (Linear discriminant analysis Effect Size). This analysis identified *Actinobacteria*, *Christensenellaceae*, and *Coriobacteriales* as strong microbial biomarkers of youth, whereas *Bacteroides* and related taxa emerged as signatures of aging (Figure 1d).

The concordance between relative abundance profiles and biomarker analysis supported the reliability of these taxonomic shifts. Together, these results demonstrated that natural aging in mice does not erode overall microbial diversity but rather reshapes gut microbial composition in a lineage‐specific manner. The consistent separation in *β*‐diversity, enrichment of specific microbial families and genera, and corroboration by LEfSe collectively highlighted a structured reorganization of the microbiome during aging, with potential functional consequences for host physiology.

### 3.2 Microbial Functional Remodeling During Aging

**FIGURE 2:**
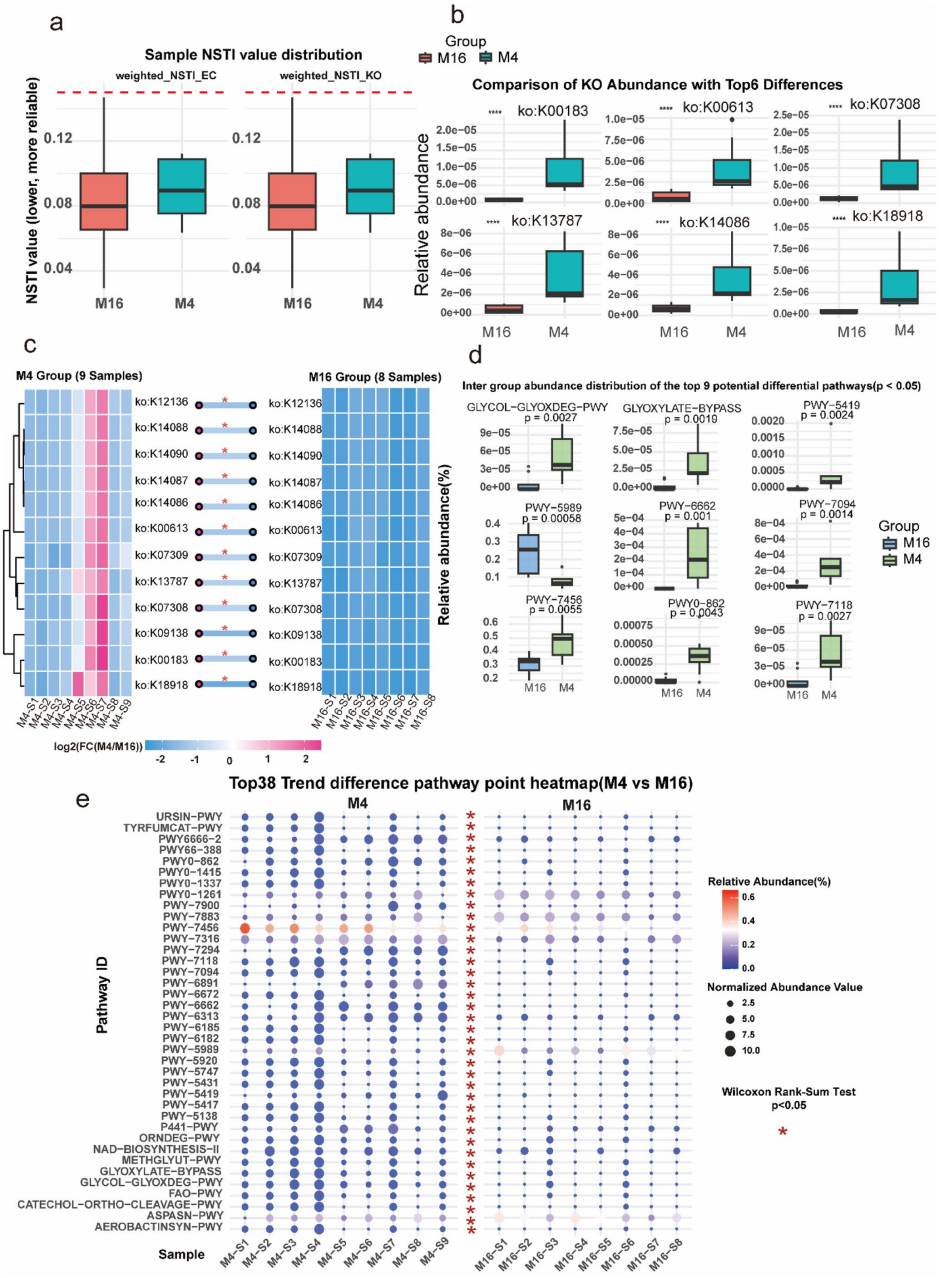
Microbial functional decline impairs redox‐supportive and energy pathways while enriching catabolic routes. (a) NSTI values for enzyme commission (EC) and KEGG ortholog (KO) predictions, all below 0.1, confirming the reliability of functional inference. (b) Differential abundance of the top six KEGG orthologs, with reduced oxidoreductases and transporters in aged mice (CM16). (c) Heatmap of KO abundance highlighting age‐dependent shifts across groups. (d) Boxplots of nine significantly altered pathways, highlighting the depletion of protective metabolic functions in CM16, including reduced NAD^+^ biosynthesis II, glyoxylate bypass, and glycolate/oxalate degradation, and tyrosine fermentation to fumarate (TYRFUMCAT‐PWY). (e) Heatmap of the top 38 differential pathways, showing broad remodeling from redox‐supportive to catabolic and pro‐inflammatory functions with age. These analyses confirmed a selective functional decline in the aged microbiota, marked by loss of protective, redox‐supportive capacity and enrichment of catabolic pathways that exacerbate systemic vulnerability.

Functional prediction revealed pronounced remodeling of microbial metabolic capacity with aging (Figure 2d,e). Importantly, NSTI values for both enzyme commission (EC) and KEGG ortholog (KO) predictions were consistently below 0.1 across all samples, supporting the high reliability of the PICRUSt2‐based inference (Figure 2a). During aging, the relative abundance of pathways that normally sustain host redox balance and energy metabolism including NAD^+^ biosynthesis, the glyoxylate bypass, and glycolate/oxalate degradation was markedly reduced (Figure 2d). Additionally, the CM16 microbiota exhibited a significant depletion of aromatic amino acid catabolism pathways, particularly tyrosine fermentation to fumarate (TYRFUMCAT‐PWY) and catechol degradation (PWY‐5419), which were significantly more abundant in young mice (Figure 2d,e). Collectively, these findings suggested that aging is characterized by a widespread loss of protective metabolic functions, including those required for the generation of beneficial indole derivatives and the maintenance of redox homeostasis.

At the gene level, KEGG ortholog analysis further highlighted widespread losses of enzymes central to energy metabolism and redox homeostasis. Notably, key enzymes including glycine amidinotransferase (K00613) were consistently diminished in aged microbiota (Figure 2b,c), underscoring impaired microbial capacity to sustain creatine biosynthesis and energy buffering. Furthermore, enzymes critical for anaerobic respiration and electron transfer, including anaerobic dimethyl sulfoxide reductase (K07308) and hydrogenase subunits (K14086), also showed depletion, further supporting a decline in the microbiota's capacity to manage redox potential and energy generation under anaerobic conditions.

A broader survey of the top 38 differential pathways revealed a consistent contraction in microbial metabolic capacity with aging. Pathways favoring protective host‐microbiota interactions, particularly those supporting energy generation (e.g., GLYOXYLATE‐BYPASS, FAO‐PWY), redox maintenance (e.g., NAD‐BIOSYNTHESIS‐II), and cellular repair, were markedly reduced in aged mice (CM16) compared to the young group (CM4) (Wilcoxon rank‐sum test, *p* < 0.05; 0.05 < FDR < 0.2). Furthermore, contrary to an expansion of pathogenic traits, pathways associated with aromatic amino acid catabolism and pro‐inflammatory processes (specifically TYRFUMCAT‐PWY, CATECHOL‐ORTHO‐CLEAVAGE‐PWY, PWY‐5419, and PWY‐5417) were also significantly diminished in the aged microbiota (Figure 2e). This simultaneous decline across diverse functional categories suggests that natural aging is characterized by a loss of metabolic potential, specifically impairing the microbiota's ability to process dietary precursors into bioactive metabolites (such as indole derivatives), rather than a selective enrichment of specific neurotoxic pathways.

### 3.3 Integration of Microbiome and Host Profiles

We integrated microbiome and metabolomic profiles to assess the relationship between microbial remodeling and host metabolic reprogramming. A moderate though statistically non‐significant, decreasing trend in indole‐3‐propionic acid (IPA) was observed in the serum and cortex of aged mice (Figure S1), which paralleled the depletion of specific IPA‐producing taxa (e.g., *Bifidobacterium*) and the significant loss of microbial aromatic amino acid catabolism pathways (e.g., TYRFUMCAT‐PWY) (Figure 2d,e). In contrast to the loss of protective functions, the aged host metabolome displayed signs of altered tryptophan metabolism (Figure 4e). While direct microbial kynurenine pathways are not primary drivers, the functional decline in microbial NAD+ biosynthesis and redox‐supportive pathways (Figure 2d) coincided with signs of systemic redox stress. This functional decline in the microbiome characterized by the loss of enzymes central to energy metabolism and redox homeostasis (Figure 2b,c), was associated with a broader pattern of metabolic drift in the host, including the depletion of circulating LPCs and altered lipid profiles in the liver and lung (Figures 4e, 5d and 6d). These findings indicate that the contraction of microbial metabolic capacity (Figure 2e) corresponds to systemic vulnerability, inferring a link between gut dysbiosis and the propagation of metabolic stress to distal organs (Figure 8a,b).

### 3.4 Microbial Metabolite Shifts Mark Early Redox Stress

**FIGURE 3:**
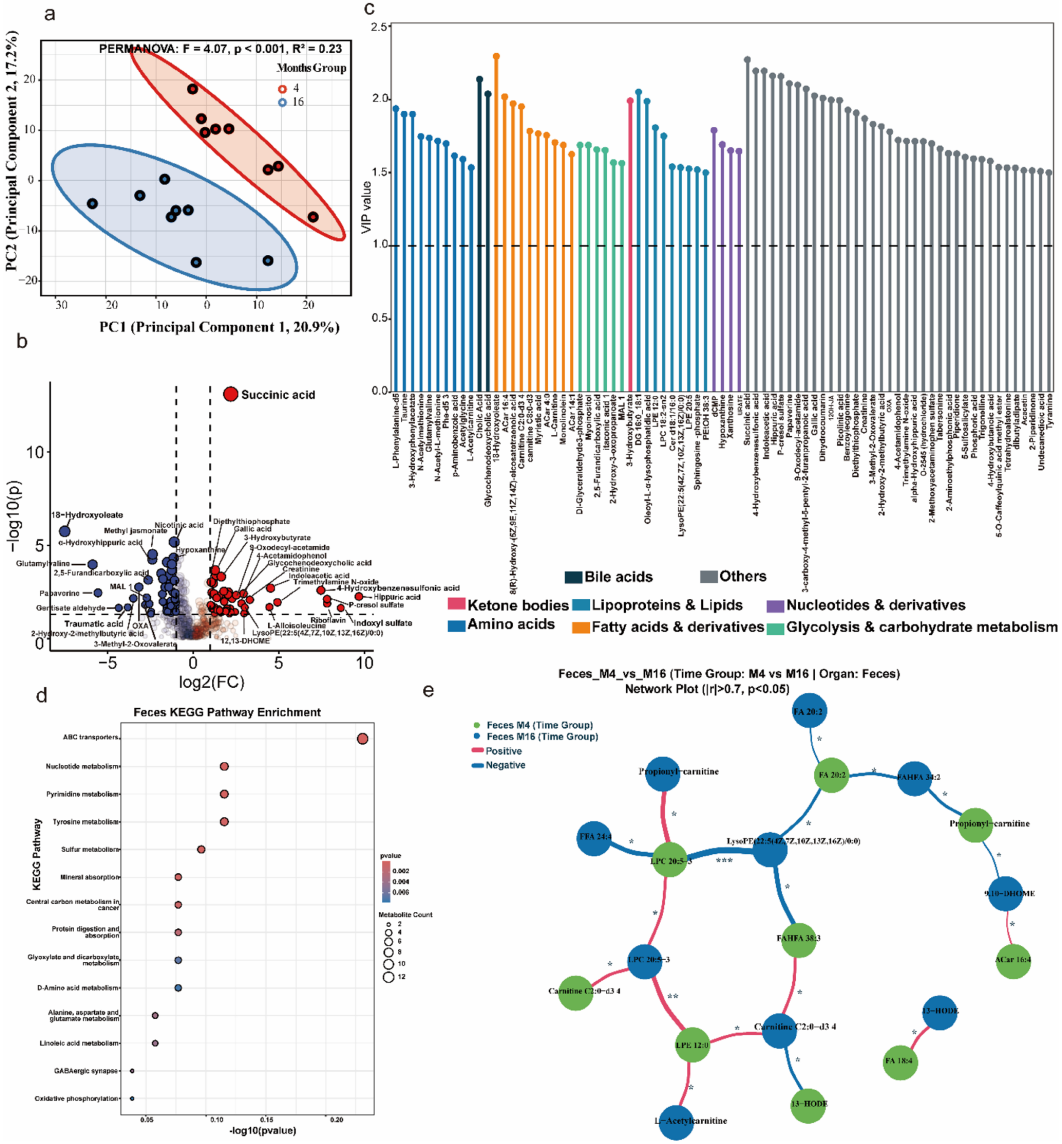
Microbial metabolite remodeling marks early systemic stress in aging. (a) Principal component analysis (PCoA) of fecal metabolomes shows clear separation between CM4 and CM16 mice (PERMANOVA, *R* ^2^ = 0.23, *p* < 0.001). (b) Volcano plot of differential fecal metabolites highlighting the accumulation of Indole‐3‐acetic acid (IAA), 3‐hydroxybutyrate, succinic acid, and trimethylamine N‐oxide (TMAO) in aged mice (CM16), while identifying metabolites enriched in young mice (CM4) on the opposing side. (c) Variable importance projection (VIP) ranking highlights top discriminatory metabolites, identifying Taurine, L‐Carnitine, and Acetylcarnitine as key markers depleted in the aged group. (d) KEGG pathway enrichment reveals significant upregulation of D‐amino acid metabolism, linoleic acid metabolism, and ABC transporter pathways in CM16. (e) Correlation network diagram shows age‐dependent rewiring of associations among LPCs, acylcarnitines, and oxidized fatty acids. Together, these results indicate that natural aging reprograms gut microbial metabolism toward a pro‐inflammatory, redox‐destabilizing profile, characterized by the enrichment of IAA and the depletion of protective carnitines and taurine.

Pseudo‐targeted fecal metabolomics revealed significant age‐associated remodeling of microbiota‐derived metabolites. Principal Component Analysis demonstrated clear separation between young (CM4) and aged (CM16) fecal metabolomes (PERMANOVA, *R* ^2^ = 0.23, *p* < 0.001; Figure 3a). Differential abundance testing identified widespread metabolic reprogramming. Consistent with the systemic accumulation of aging‐related metabolites, we observed that Succinic acid, Hippuric acid, and Trimethylamine N‐oxide (TMAO) were significantly enriched in the aged group (Figure 3b; Figure S1). This accumulation was accompanied by specific shifts in tryptophan and lipid metabolism. Indole‐3‐acetic acid (IAA) was significantly elevated (*q* < 0.05, Fold‐Change > 20), alongside 3‐hydroxybutyrate, suggesting a shift toward auxin derivatives and ketogenesis. In terms of protective capacity, we observed a significant downregulation of antioxidant amino acids and mitochondrial cofactors, specifically Taurine (VIP > 1.8), rather than a statistically significant loss of IPA (VIP < 1) (Figure 3b,c). Variable importance projection (VIP) analysis ranked these discriminatory metabolites spanning amino acids, fatty acids, and nucleotide derivatives, underscoring broad biochemical reprogramming (VIP > 1.0; Figure 3c).

**FIGURE 4:**
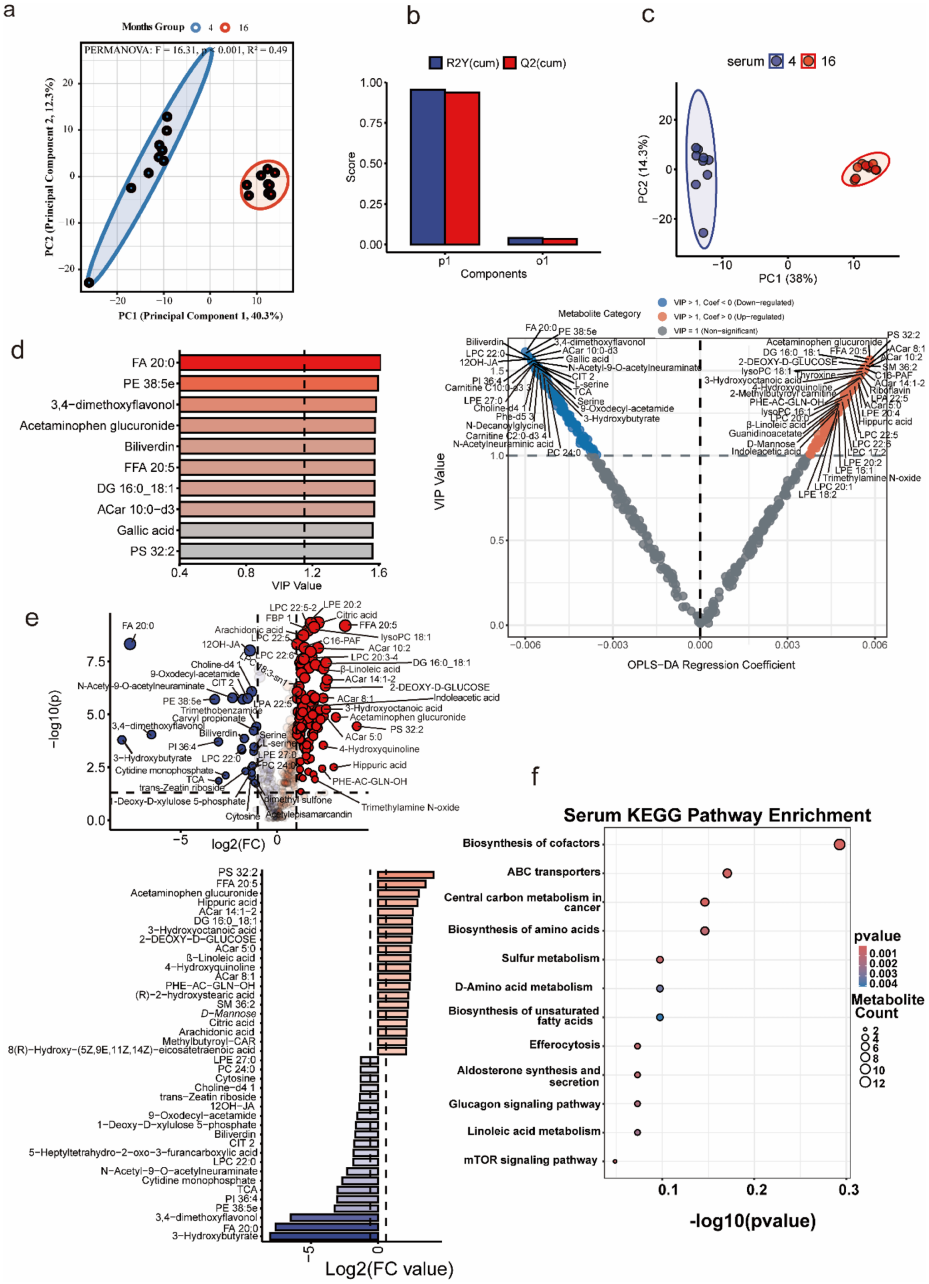
Circulating lipids and microbial metabolites reflect systemic redox collapse. (a–c) PCA and OPLS‐DA analyses show clear separation of young (CM4, blue) and aged (CM16, red) serum profiles. (d) Combined Variable Importance in Projection (VIP) scores and OPLS‐DA coefficient S‐plot highlighting top discriminant metabolites, identifying FA 20:0 and Biliverdin as key markers of youth (Blue), and Acylcarnitines as drivers of the aged phenotype (Red). (e) Combined Volcano plot and Log2 fold‐change (Log2FC) analysis showing the significant depletion of FA 20:0, PE 38:5e, and Biliverdin, alongside the enrichment of Free Fatty Acids (FFA 20:5) and oxidized metabolites in aged serum. (f) KEGG pathway enrichment demonstrates significant alterations in the biosynthesis of cofactors, sulfur metabolism, D‐amino acid metabolism, and ABC transporters. Together, these findings indicate that circulating lipids and redox‐related metabolites are strongly associated with the systemic propagation of metabolic stress during aging.

KEGG pathway enrichment revealed that CM16 metabolomes were significantly enriched in D‐Amino acid metabolism, linoleic acid metabolism, and ABC transporter pathways (FDR < 0.1; Figure 3d), consistent with a shift toward energy‐imbalanced metabolism. Correlation network analysis further showed extensive rewiring of metabolite‐metabolite associations, indicating disrupted lipid remodeling and redox balance in the intestinal milieu (Figure 3e). These findings demonstrated that aging is associated with microbial metabolite remodeling from a protective, homeostatic profile (dominated by taurine, carnitines, and succinate) to a distinct catabolic state. This intestinal shift is associated with nascent systemic metabolic stress. While 16‐month‐old mice do not yet exhibit the severe frailty of advanced age, our results demonstrate clear perturbations in redox‐sensitive metabolites concurrent with a ~40%–60% decline in serum LPCs and IPA (Figure 4d,e). This suggests that metabolic reprogramming precedes overt physiological decline, positioning these shifts as early markers of systemic aging.

### 3.5 Circulating Fingerprint: Serum Lipids and Metabolites Index Systemic Aging

Circulating metabolites revealed a robust systemic aging fingerprint, with multivariate analyses confirming clear metabolic divergence between CM4 and CM16 mice (Figure 4a–c). The most striking signature was the differential regulation of lipid species. While specific saturated fatty acids (e.g., FA 20:0) and phosphatidylethanolamines (PE 38:5e) were significantly depleted in aged serum (Log2FC < −2), other lipid classes showed marked accumulation (Figure 4d,e). Contrary to the generic LPC depletion model, we observed a specific loss of LPC 22:0, whereas other species like LysoPC 18:1 were elevated (Figure 4e; Figure S2). Importantly, the aged circulating environment was characterized by a metabolic signature of redox stress, defined by the specific loss of antioxidant capacity and the accumulation of mitochondrial stress markers. Biliverdin, a potent antioxidant bile pigment, was significantly reduced in aged serum (Figure 4d). Conversely, markers of incomplete beta‐oxidation and oxidative stress, including Free Fatty Acids (FFA 20:5), Acylcarnitines (ACar 5:0, 8:1), and Trimethylamine N‐oxide (TMAO), were significantly enriched (Figure 4e).

Quantitative fold‐change analysis indicated profound shifts: FA 20:0 and Biliverdin declined by > 80% (Log2FC ~−3 to −5), whereas FFA 20:5 and ACar species increased by > 50% (all FDR < 0.05). Pathway enrichment analysis mapped these shifts to Biosynthesis of cofactors, ABC transporters, and Sulfur metabolism, showing the strongest perturbations in aged serum (Figure 4f). Collectively, these results position the serum metabolome not just as a passive carrier, but as an active reflector of mitochondrial dysfunction (acylcarnitine accumulation) and redox failure (biliverdin depletion), linking gut‐derived stress (TMAO) to systemic metabolic aging.

### 3.6 Hepatic Lipid Accumulation and Metabolic Remodeling

**FIGURE 5:**
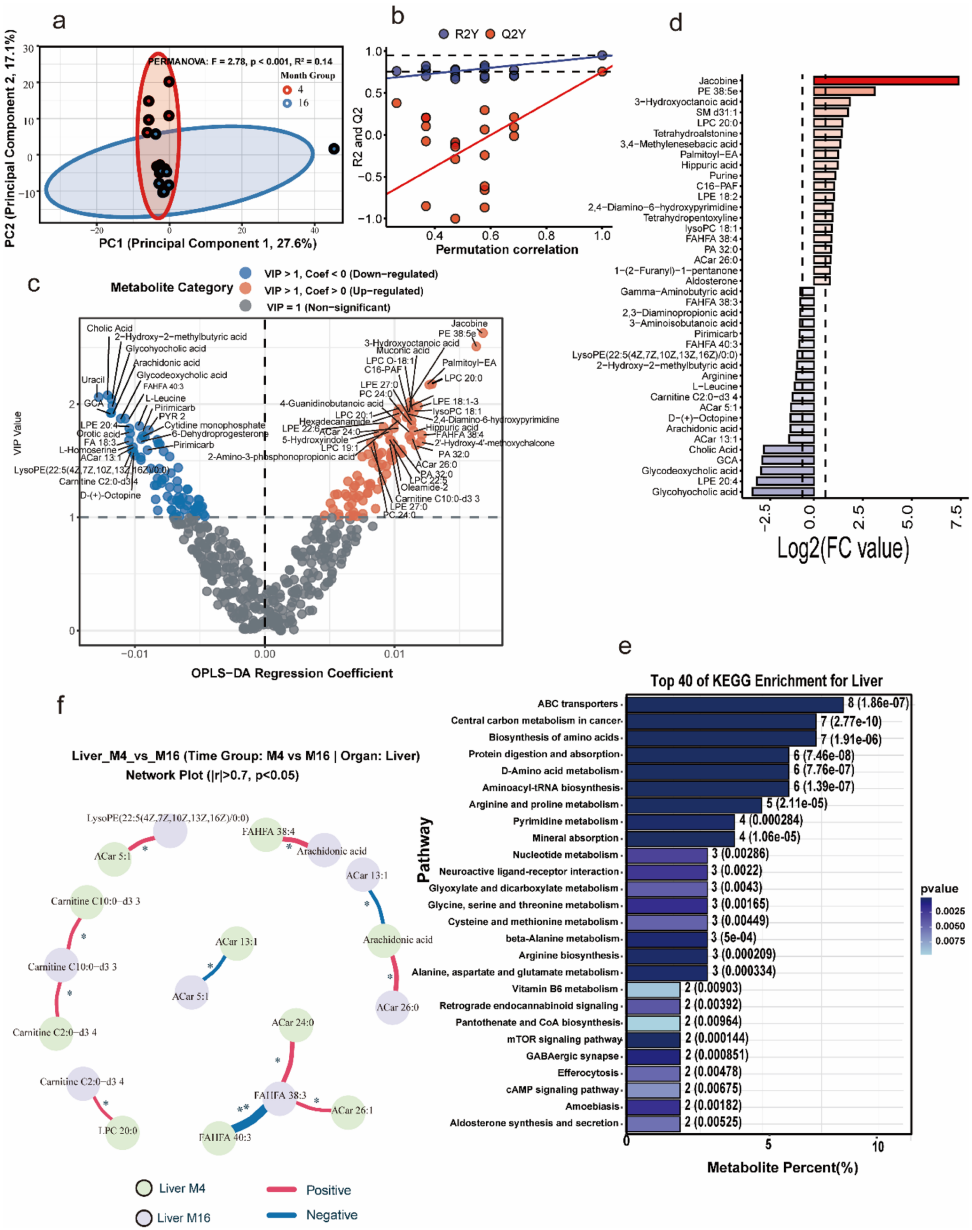
Hepatic metabolome exhibits ferroptosis‐like remodeling and metabolic network collapse. (a) Principal component analysis (PCoA) reveals separation of CM4 and CM16 liver metabolomes (PERMANOVA, *R* ^2^ = 0.14, *p* < 0.001). (b) OPLS‐DA permutation validation confirms model robustness. (c) Combined VIP and OPLS‐DA S‐plot identifies key discriminant metabolites. (d) Log2 fold‐change analysis shows the enrichment of Lysophosphatidylcholines (LPC 20:0) and Phosphatidylethanolamines (PE 38:5e), alongside the depletion of Arachidonic acid, Acylcarnitines (ACar 5:1, 13:1), and Bile acids (Glycocholic acid) in aged livers. (e) KEGG pathway enrichment indicates alterations in ABC transporters, Biosynthesis of amino acids, and Central carbon metabolism. (f) Metabolite correlation network highlights disrupted coordination among LPCs, acylcarnitines, and fatty acids. Supplementary analyses show that liver‐serum metabolic coupling is weakened in CM16, underscoring the systemic collapse of lipid‐redox homeostasis.

Consistent with the systemic aging trajectory, the liver metabolome exhibited a profound reorganization. However, distinct from the depletion patterns observed in serum, the aged liver was characterized by a specific accumulation of lipid species. Differential metabolite analysis demonstrated that aged livers were significantly enriched in Lysophosphatidylcholines (LPCs) (including LPC 20:0, LPC 20:1, LPC 22:5) and Phosphatidylethanolamines (e.g., PE 38:5e) (Figure 5c,d). This enrichment contrasts with the critical depletion of Arachidonic acid, Glycocholic acid, and mitochondrial Acylcarnitines (ACar 5:1, ACar 13:1), which were identified as key markers of the youthful liver phenotype (Figure 5d, Blue Bars). KEGG pathway analysis mapped these alterations to ABC transporters, Central carbon metabolism, Biosynthesis of amino acids, and Protein digestion and absorption, implicating a shift in hepatic transport and nitrogen balance rather than oxidative phosphorylation (Figure 5e).

Correlation network analysis examining the associations between lipid species and acylcarnitines revealed that aging disrupts the coordinated interactions required for homeostasis. In young mice, a coherent network linked Arachidonic acid to mitochondrial carriers (ACar 26:0, ACar 13:1) (Figure 5f). However, in aged mice, this network shifts toward a dominance of accumulated LPC species (e.g., LPC 20:0) and FAHFAs, suggesting that hepatic aging is defined by a failure to mobilize lipids effectively, leading to their retention and a concurrent loss of essential PUFAs and mitochondrial capacity.

### 3.7 Pulmonary Immune‐Redox Activation

**FIGURE 6:**
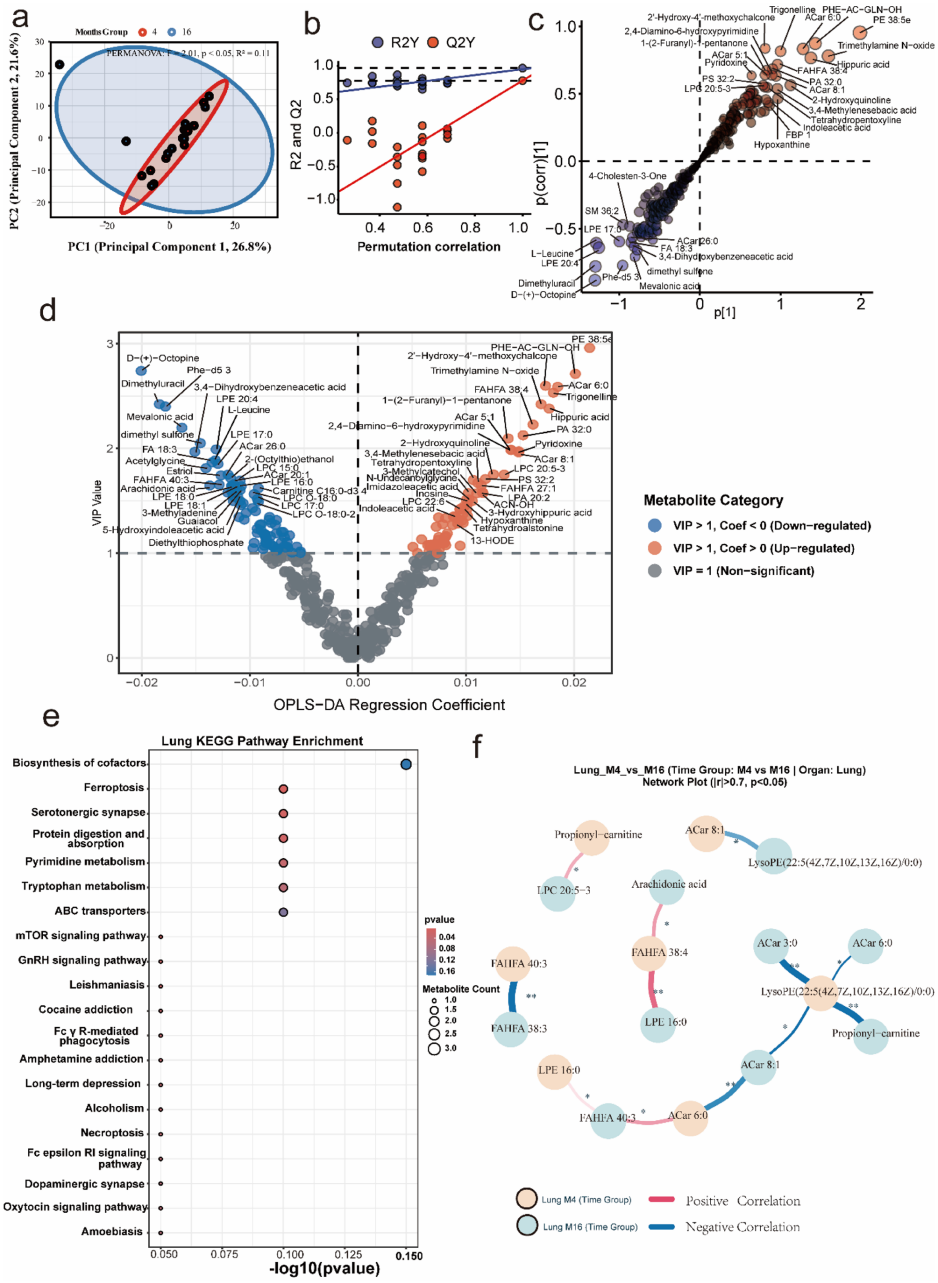
Lung metabolome remodeling reflects oxidative infiltration and immune‐redox activation. (a) PCA shows separation of CM4 and CM16 lung metabolomes (PERMANOVA, *R* ^*2*^ = 0.11, *p* < 0.05). (b) OPLS‐DA permutation test confirms model stability. (c) OPLS‐DA correlation plot highlights discriminant metabolites. (d) Combined VIP and OPLS‐DA regression coefficient plot identifies the upregulation of Acylcarnitines (ACar 5:1, 6:0), Hippuric acid, and TMAO in aged lungs, alongside the depletion of PUFAs (Arachidonic acid, FA 18:3) and detoxification markers (Dimethyl sulfone, Octopine). (e) KEGG enrichment reveals ferroptosis‐like remodeling, tryptophan metabolism, ABC transporters, pyrimidine metabolism, serotonergic synapse, and Fcγ receptor‐mediated phagocytosis as significantly impacted pathways. (f) Metabolite correlation network demonstrates age‐dependent loss of coordinated interactions among LPCs, FAHFAs, and acylcarnitines.

Despite not being a classical metabolic organ, the aged lung metabolome displayed pronounced age‐associated remodeling. Such tissue remodeling is often regulated by specific phosphorylation events in the Smad signaling pathway that promote fibrosis (Yu et al. 2025). Multivariate analyses revealed clear separation between CM4 and CM16 groups (PERMANOVA, *R* ^*2*^ = 0.11, *p* < 0.05), validated by robust OPLS‐DA permutation performance (Figure 6a,b). Differential metabolite analysis showed that aged lungs accumulated hippurate, trigonelline, and trimethylamine N‐oxide (TMAO), alongside reductions in polyunsaturated fatty acids (PUFAs) and detoxification‐associated metabolites including dimethyluracil, dimethyl sulfone, and D‐(+)‐Octopine (Figure 6c,d). These signatures suggested a transition to a redox‐impaired and detoxification‐deficient pulmonary environment, coinciding with infiltration of gut‐derived microbial catabolites such as TMAO and indole‐3‐acetic acid. KEGG enrichment highlighted ferroptosis‐like remodeling, pyrimidine metabolism, ABC transporters, tryptophan metabolism, and Fcγ receptor‐mediated phagocytosis as significantly altered pathways (Figure 6e).

This pointed to converging ferroptosis‐like stress, impaired nucleotide salvage, and chronic immune activation as hallmarks of the aged lung. Network analysis revealed disrupted interactions among LPCs, acylcarnitines, and fatty acid esters of hydroxy fatty acids (FAHFAs) in CM16, indicating breakdown of protective lipid‐redox coordination (Figure 6f). Supplementary cross‐organ correlation analyses showed that while young mice maintained coherent lung‐serum lipid coupling, aged mice exhibited fragmented and weakened associations (Figure S4). These findings identified the lung as a metabolically vulnerable site during aging, where ferroptosis‐like remodeling, immune‐redox activation, detoxification failure, and infiltration of microbial catabolites are associated with compromised pulmonary resilience and systemic stress.

### 3.8 Cortical Neurochemical Imbalance and Barrier Dysfunction

**FIGURE 7:**
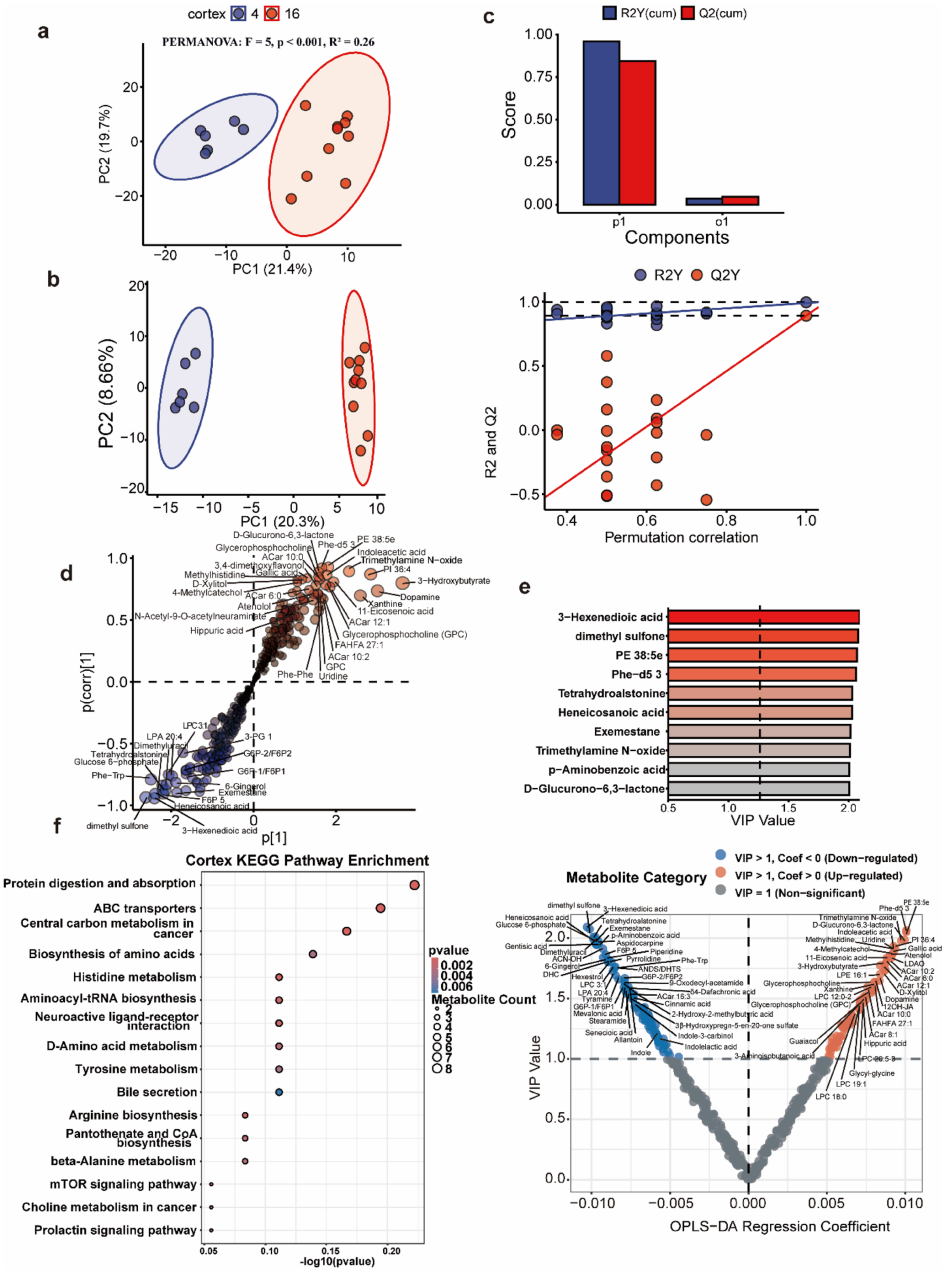
Cortical metabolome reveals neurochemical imbalance and loss of systemic metabolic integration. (a, b) PCA demonstrates strong separation of CM4 and CM16 cortical metabolomes (PERMANOVA, *R* ^2^ = 0.26, *p* < 0.001). (c) Combined OPLS‐DA model summary (R2Y, Q2) and permutation test results confirming model robustness. (d) OPLS‐DA correlation S‐plot identifies discriminant metabolites, including the upregulation of TMAO, dopamine, and PE 38:5e, and the downregulation of dimethyl sulfone and 3‐hexenedioic acid. (e) Combined Variable Importance in Projection (VIP) scores and OPLS‐DA regression coefficient plot highlighting key contributors to age‐associated cortical differences. (f) KEGG pathway enrichment reveals disruptions in Protein digestion and absorption, ABC transporters, Central carbon metabolism, and Biosynthesis of amino acids. Supplementary analyses show that strong cortex–serum metabolic coupling in CM4 becomes fragmented in CM16, underscoring the loss of systemic integration and enhanced neurochemical vulnerability.

The cortex exhibited the most pronounced and functionally disruptive metabolic aging signature. Principal Component analysis demonstrated strong separation between CM4 and CM16 cortical metabolomes (PERMANOVA, *R* ^*2*^ = 0.26, *p* < 0.001), validated by robust OPLS‐DA model performance (Figure 7a–c). Differential metabolite analysis revealed significant enrichment of gut‐ and liver‐derived catabolites, including trimethylamine N‐oxide (TMAO) and indole‐3‐acetic acid (IAA), alongside dopamine, hippurate, and phosphatidylethanolamines (e.g., PE 38:5e) in aged cortices, beside depletion of protective redox‐sensitive metabolites such as dimethyl sulfone, 3‐hexenedioic acid, and key phospholipids (Figure 7d,e). The detection of these normally gut‐restricted or highly metabolized compounds in the cortex provided inferential evidence of age‐related loss of Blood–Brain Barrier selectivity/integrity, a key indicator of neurovascular vulnerability.

KEGG pathway enrichment highlighted altered amino acid metabolism (histidine, tyrosine, arginine), protein digestion and absorption, ABC transporter activity, and neuroactive ligand‐receptor signaling, suggesting broad dysregulation of neurotransmission, nutrient handling, and membrane dynamics (Figure 7f). Cross‐organ correlation analyses further revealed that while young cortices maintained coherent cortex‐serum coupling dominated by LPC‐carnitine and PUFA‐FAHFA associations, aged cortices exhibited fragmented and weakened correlations (Figure S5). These results suggested that natural aging is associated with changes in the cortical metabolic environment, including excitatory imbalance, ferroptosis‐like lipid remodeling, and loss of systemic metabolic integration, consistent with neurochemical vulnerability.

### 3.9 Multi‐Organ Integration Defines Conserved Metabolic Aging Axes

**FIGURE 8:**
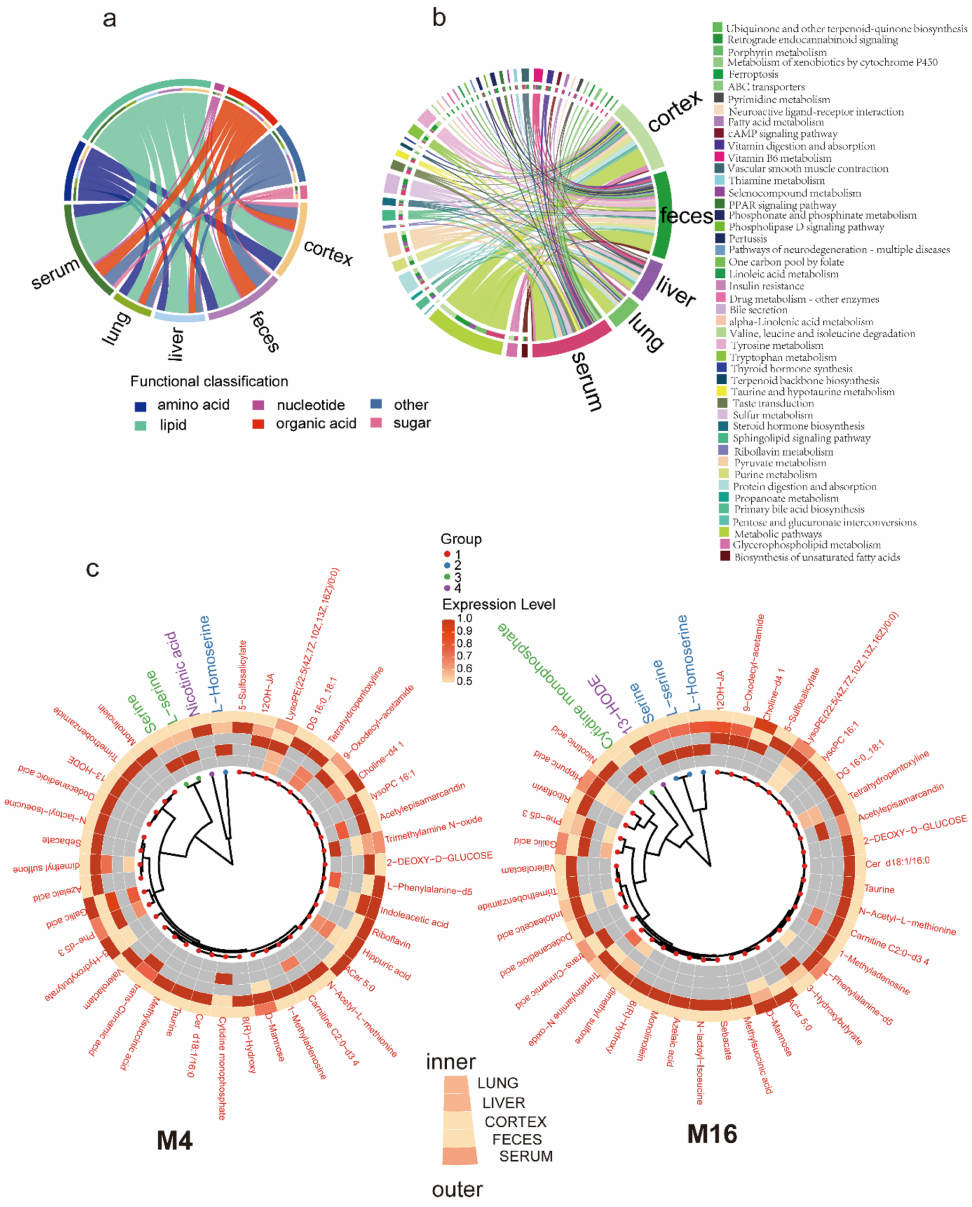
Cross‐tissue mapping of metabolite classes and pathways during aging. (a) Chord diagram of metabolite class flows across feces, serum, liver, lung, and cortex, grouped by functional categories (amino acids, lipids, organic acids, sugars). (b) Chord diagram of KEGG pathway enrichment linking tissue‐specific alterations to conserved aging signatures, including ABC transporter activity, biosynthesis of unsaturated fatty acids, and ferroptosis. (c) Circular hierarchical clustering heatmaps of differential metabolites for the CM4 (Young) and CM16 (Aged) groups, visualizing the distinct clustering patterns and expression level differences of metabolites within each age group. Together, these analyses highlight the age‐dependent rewiring of metabolic networks and the tissue‐specific accumulation of stress markers.

Despite organ‐specific differences, integrative analysis identified a coherent and conserved aging mechanism associated with changes from the gut into systemic circulation and peripheral tissues. Rather than a uniform ferroptosis signature, the ABC Transporter and Tryptophan/Indole axes were identified as the dominant conserved systemic disruptions, appearing significantly enriched across fecal, hepatic, pulmonary, and cortical datasets. Chord diagram visualization highlighted metabolite class distributions across feces, serum, liver, lung, and cortex (Figure 8a). Amino acids, lipids, and organic acids dominated cross‐compartment flux, with microbial‐derived metabolites (e.g., tryptophan catabolites) serving as key connectors between the gut and distal organs.

Pathway‐level integration further revealed both conserved and tissue‐specific signatures (Figure 8b). ABC transporters and Amino acid biosynthesis were preferentially enriched in the liver (Figure 5e), consistent with its role in nutrient processing and detoxification. In contrast, Ferroptosis and Immune‐redox pathways (e.g., Fcγ receptor‐mediated phagocytosis) were specifically enriched in the lung (Figures 6e and 8b), providing *in silico* evidence of pulmonary‐specific vulnerability to oxidative cell death. The cortex displayed unique enrichment in Neuroactive ligand‐receptor interaction and Protein digestion pathways (Figure 7f). Circular clustering heatmaps of differential metabolites underscored these tissue‐dependent profiles (Figure 8c). While specific lipids like LPCs showed divergent trends (depleted in serum, accumulated in liver), pro‐oxidative signaling molecules showed systemic propagation: Trimethylamine N‐oxide (TMAO) was consistently upregulated across serum, lung, and cortex (Figures 4e, 6d and 7d), highlighting it as a robust systemic marker of the aged metabolic footprint.

**FIGURE 9:**
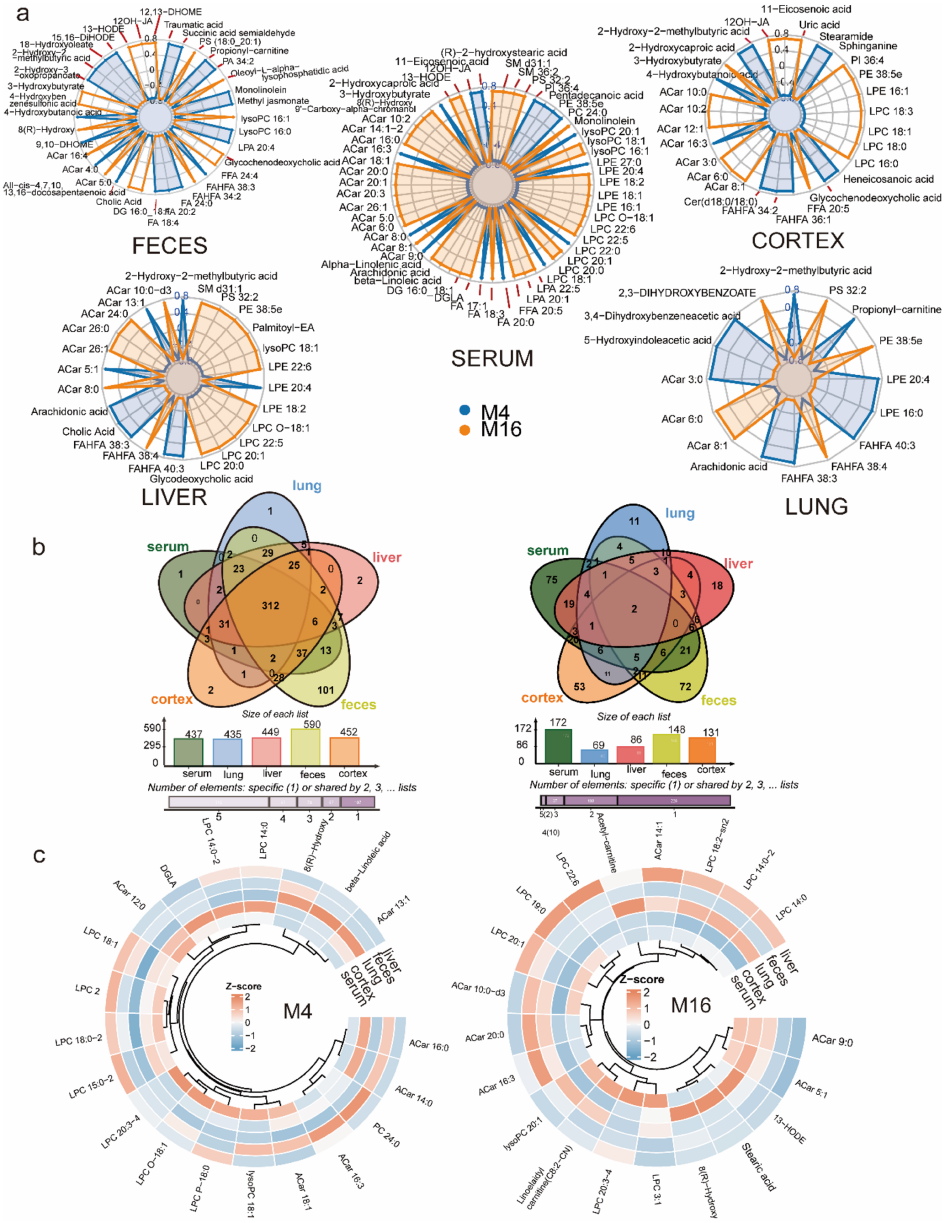
Quantitative comparisons emphasize organ‐specific metabolic vulnerabilities. (a) Radar plots showing the distribution of metabolite classes across organs, highlighting the distinct lipid‐heavy profile of the liver and the microbial‐dominated profile of feces. (b) Venn diagrams showing the overlap of Total Detected Metabolites (Left) and Differentially Expressed Metabolites (Right). The low overlap in the right panel indicates that metabolic aging is characterized primarily by tissue‐specific alterations rather than a uniform systemic signature. (c) Circular *Z*‐score clustering of metabolites in CM4 (left) and CM16 (right) groups, illustrating the distinct expression patterns of LPCs and Acylcarnitines across the serum, liver, and cortex.

Quantitative comparisons emphasized distinct organ‐specific metabolic footprints (Figure 9a). Radar plots demonstrated that while the feces carried the dominant microbial signatures (enriched in bile acids and fatty acids), the cortex and liver exhibited highly specialized metabolic profiles, with the liver showing pronounced lipid remodeling and the cortex enriched in neuroactive metabolites. Venn analysis clarified the extent of this specificity (Figure 9b). While the global metabolome coverage (Left Venn) showed a large overlap of detected species, the differential analysis (Right Venn) revealed highly tissue‐specific aging responses. The majority of significantly altered metabolites were unique to a single tissue (e.g., 75 specific to serum, 72 to feces, 53 to cortex), with only a small subset shared across multiple organs. This highlights that while aging is a systemic event, the metabolic response is largely determined by the local tissue environment rather than a uniform systemic shift. Circular *Z*‐score clustering (Figure 9c) further illustrated these divergent remodeling patterns. Contrary to a synchronized systemic depletion, LPCs and Acylcarnitines displayed tissue‐dependent directionality: while previous analyses indicated LPC accumulation in the liver (Figure 5d), the *Z*‐score maps highlight that these lipid classes undergo distinct regulatory shifts in the serum and cortex. This underscores that metabolic aging is defined by the loss of inter‐organ coordination, where the liver may accumulate lipids that the serum fails to distribute, rather than a simple unidirectional depletion across the body.

### 3.10 Tryptophan Catabolism and Indole Pathways Mediate Inflammaging

Integration of microbiome functional shifts with host metabolomics highlighted tryptophan metabolism as a conserved aging mechanism. Rather than a simple depletion of neuroprotective factors, we observed a shift toward the accumulation of specific microbial catabolites. While Indole‐3‐propionic acid (IPA) showed a decreasing trend (non‐significant), pro‐oxidative and signaling metabolites, including Indole‐3‐acetic acid (IAA) and Indolelactic acid, were significantly accumulated across the gut‐brain axis. In the cortex and serum, this remodeling was characterized by the upregulation of Indole‐3‐acetic acid (IAA) and Trimethylamine N‐oxide (TMAO) (Figures 4d and 7d), suggesting that the aged blood–brain barrier is permeable to these gut‐derived signaling molecules. Multi‐organ profiling confirmed that the systemic accumulation of TMAO and the coordinated dysregulation of LPCs occurred concurrently across the gut, serum, liver, lung, and cortex, reinforcing the systemic propagation of metabolic imbalance.

### 3.11 Quantitative Validation of Key Biomarkers via Meta‐Analysis

Our systematic literature review identified 40 studies providing complementary evidence for the mechanistic link between gut microbiota alterations and systemic aging phenotypes. These studies were categorized into natural aging baseline profiling (*n* = 18), Fecal Microbiota Transplantation (FMT) interventions (*n* = 9), and probiotic supplementation strategies (*n* = 23). Profiling of natural aging models (*n* = 18) confirmed profound systemic degradation, characterized by the consistent accumulation of inflammatory factors (IL‐6, IL‐1β, TNF‐α) and oxidative stress markers (MDA, p16) across the serum, colon, liver, and brain, alongside a marked depletion of barrier indicators (ZO‐1, MUC2, Ocln1) and antioxidant capacity (SOD, GSH). Meta‐analysis of interventional studies demonstrated significant therapeutic effects of gut microbiota manipulation on these aging‐related outcomes. FMT studies (*n* = 9) revealed that transplanting microbiota from young donors into aged recipients effectively reversed the hallmarks of gut barrier decline, significantly upregulating tight junction proteins (ZO‐1, Cldn1, Ocln1) and mucin (MUC2) in the intestine, while concurrently lowering systemic endotoxemia and circulating I‐FABP levels. Furthermore, young‐to‐old FMT suppressed neuroinflammation, reducing IL‐6, IL‐1β, and TNF‐α expression in both the colon and the brain.

Complementing the FMT data, probiotic supplementation studies (*n* = 23) deploying strains such as *Lactobacillus plantarum* , *Bifidobacterium animalis* , and *Lacticaseibacillus paracasei* provided robust evidence for microbiota‐mediated redox buffering. Probiotic interventions in aged mice consistently mitigated oxidative stress by significantly decreasing malondialdehyde (MDA) accumulation and restoring superoxide dismutase (SOD) and glutathione (GSH) levels across the liver, brain, and serum. Together, this quantitative synthesis confirms that modulating the gut microbiota either through whole‐community transfer or targeted probiotic supplementation can causally intercept the systemic inflammatory and ferroptosis‐like oxidative cascades identified in our multi‐organ atlas.

## 4 Discussion

Aging is increasingly recognized as both a systemic process and a tissue‐specific degeneration orchestrated by conserved biochemical stressors, including microbial metabolites and redox imbalance (López‐Otín et al. 2023). Our multi‐organ metabolomic atlas revealed associations across the gut‐serum‐liver‐lung‐cortex axis, defining the molecular fingerprint of mid‐life metabolic aging. This molecular fingerprint of mid‐life metabolic aging is defined by convergence on tryptophan catabolism, LPC homeostasis, and ferroptosis‐like signaling, suggesting a critical pathway for systemic redox vulnerability (Hamidu et al. 2025; Wu et al. 2021).

### 4.1 The Gut Microbiota as an Active, Upstream Driver of Metabolic Drift

The gut microbiome has emerged as a key regulator of host physiology during aging, holding potential as both an ideal aging‐related biomarker and an intervention target. Our study builds upon this concept by identifying a conserved metabolic aging signature propagated from the gut. Recent reviews emphasized that microbial biomarkers can monitor the efficacy of interventions (Xu et al. 2024), a paradigm our multi‐omics atlas directly advanced by nominating specific microbial taxa and their metabolic outputs as tractable targets. Our findings revealed that natural aging in mice is not characterized by a system‐wide loss of microbial diversity, but by a selective compositional and functional remodeling. Consistent with the LEfSe analysis (Figure 1d), our aged mice exhibited an enrichment of *Bacteroides* and *Rikenellacea*e, accompanied by a depletion of beneficial lineages such as *Christensenellaceae*, *Coriobacteriaceae*, and *Faecalibaculum*. These compositional changes aligned with predictive metagenomics (NSTI < 0.1), which indicated a broad loss of metabolic capacity (Niu et al. 2025; Wu et al. 2025). Specifically, pathways that normally sustain host redox balance, including NAD biosynthesis, the glyoxylate bypass, and even aromatic amino acid catabolism, were significantly reduced in the aged microbiome (Figure 2d).

Despite this functional decline in the gut, the host accumulated pro‐oxidative metabolites, suggesting a failure of containment or clearance. The accumulation of Indole‐3‐acetic acid (IAA), Indolelactic acid, and TMAO in the serum and cortex (Figures 4e and 7d) in the face of reduced microbial production capacity points toward increased gut permeability and compromised hepatic detoxification as the primary drivers of this systemic toxic indole shift (File S1).

### 4.2 IAA, TMAO, and Lipid Dysregulation: Propagating Redox Stress to Circulation

Integration of microbiome profiles with serum metabolomics demonstrated that microbial remodeling directly shaped circulating metabolites. Rather than a simple depletion of neuroprotective factors, we observed a shift toward the accumulation of specific pro‐oxidative signals. While Indole‐3‐propionic acid (IPA) showed a decreasing trend, the most robust signature was the significant accumulation of Indole‐3‐acetic acid (IAA) and Trimethylamine N‐oxide (TMAO) in the serum and cortex (Figures 4e and 7d), metabolites previously implicated in endothelial dysfunction and inflammaging (Kurhaluk et al. 2025). In parallel, the serum exhibited a consistent depletion of specific lysophosphatidylcholines (e.g., LPC 22:0) and long‐chain fatty acids (FA 20:0), alongside the loss of the endogenous antioxidant Biliverdin (Figure 4d). This contrasts with the enrichment of oxidized lipid products and acylcarnitines, suggesting a systemic failure in lipid‐redox buffering. These findings highlight the gut microbiota not merely as a passive observer, but as a critical origin of aging‐associated metabolite imbalances, where the production of TMAO and IAA exacerbates systemic vulnerability. Restoring beneficial taxa could mitigate this mechanism; natural compounds like essential oils and tannins have demonstrated efficacy in improving blood antioxidation by reshaping the fecal microflora (Chen et al. 2023).

### 4.3 Disrupted Lipid Homeostasis and Pulmonary Ferroptosis Vulnerability

A central finding of this study is that microbial remodeling is associated with profound, yet tissue‐specific, changes in lipid metabolism during aging. Rather than a uniform systemic depletion, our data indicated a dysregulation of lipid distribution. Aged mice exhibited a significant depletion of protective LPCs in the serum (Figure 4e), which contrasted with their accumulation in the liver (Figure 5d). This pattern coupled with the enrichment of ABC transporter pathways in the liver (Figure 5e) suggests that aging is characterized by a failure in hepatic lipid mobilization, leading to retention in the liver and deficit in the circulation (Parks et al. 2012). While this lipid imbalance was systemic, the susceptibility to ferroptosis‐like remodeling appeared most critical in the lung, where the pathway was specifically enriched (Figure 6e). This fundamentally moves the paradigm from widespread oxidative stress to a defined, organ‐specific mechanism of vulnerability (Han et al. 2024). LPCs are increasingly recognized as signaling molecules that maintain membrane integrity; their decline in serum has been associated with immune‐metabolic dysregulation (Law et al. 2019).

Our data extends these associations by suggesting that microbial functional decline (e.g., reduced energy metabolism) may be linked to this blockade in lipid transport. In the cortex, this cascade culminated not in ferroptosis, but in the accumulation of TMAO and neuroactive ligands (Figure 7d), features consistent with blood–brain barrier dysfunction and neurochemical imbalance (Wang et al. 2025). Thus, the systemic dysregulation of lipids observed across multiple organs is a critical pathway influencing the trajectory toward tissue‐specific vulnerabilities: ferroptosis in the lung, steatosis‐like retention in the liver, and toxic metabolite accumulation in the brain (File S1).

### 4.4 Toward a Systems Atlas of Natural Aging

The strength of our study lies in its integrative, multi‐organ design, which captures the systemic handoff of metabolites across organ systems. Unlike previous single‐tissue approaches, our work constructed a detailed mechanistic framework associating local microbiome changes with systemic aging phenotypes. Specifically, we identified a divergent pattern of lipid remodeling characterized by the failure of hepatic lipid mobilization (LPC accumulation) and subsequent serum depletion, alongside the convergent accumulation of pro‐oxidative microbial metabolites (TMAO, IAA) across the gut‐brain axis (Zhang, Wu, et al. 2025). Our cohorts represented natural mid‐life aging under controlled SPF conditions, thereby defining an endogenous trajectory of metabolic decline. Profiling this aged time point is a major strength, as it uniquely captures the early metabolic vulnerabilities initiated by microbial decline before overt late‐life organ failure. By systemically connecting microbial dysbiosis to circulating metabolite drift, we have constructed a framework for how local microbiome changes are associated with the systemic propagation of redox stress and lipid transport failure. This atlas underscores the gut microbiota as a correlate strongly associated with metabolic reprogramming in liver, lung, and cortical physiology.

While this study provided a correlative multi‐organ atlas, the notion that gut microbial remodeling influences host metabolic aging is well supported by the broader literature. For instance, fecal microbiota transplantation (FMT) from young donors to aged recipients has been demonstrated to restore a more youthful gut community and ameliorate aging‐related impairments (Xu et al. 2024). This positions the gut microbiome as a modifiable factor that may influence host health during aging. Targeting the gut barrier remains a viable strategy for delaying these systemic drifts; interventions such as Artemisia argyi or Astragalus have been shown to improve intestinal barrier function by acting on specific microbiota (Chen et al. 2022; Su et al. 2023).

### 4.5 Integration With Meta‐Analysis Findings

While our multi‐organ atlas provided a correlative framework for the systemic handoff of metabolites, the notion that gut microbial remodeling causally influences host metabolic aging is strongly supported by our integrated meta‐analysis of 40 independent studies. Our atlas identified a conserved trajectory of systemic redox vulnerability and compromised barrier integrity. The meta‐analysis directly validates this by demonstrating that replacing the aged microbiome via FMT from young donors successfully restores intestinal barrier indicators (ZO‐1, MUC2, Ocln1) and reduces systemic endotoxin leakage. Furthermore, targeted probiotic interventions across 23 studies consistently reversed the specific oxidative signatures we observed, effectively lowering MDA levels and restoring endogenous antioxidant capacity (SOD, GSH) in distal organs such as the liver and brain. This positions the gut microbiome not merely as an associative biomarker, but as a functionally validated, modifiable upstream driver capable of reversing systemic inflammaging and oxidative decline.

### 4.6 Human‐Parallel Biomarkers in a Systemic Context

Our study identified several biomarkers that parallel metabolic shifts reported in human aging cohorts. While Indole‐3‐propionic acid (IPA) showed only a decreasing trend (non‐significant), the accumulation of Indole‐3‐acetic acid (IAA) and Trimethylamine N‐oxide (TMAO) was robust across the serum and cortex of aged mice (Figures 4e and 7d). This directly mirrors human data linking elevated TMAO to vascular inflammation and cognitive decline. Similarly, the serum depletion of LPCs (e.g., LPC 22:0) observed in our mice parallels findings in human plasma, where declines in specific lysophosphatidylcholines predict frailty and impaired mitochondrial capacity (Kim et al. 2023; Semba et al. 2019). Instead of the Kynurenine/Tryptophan ratio, which showed high variability, we observed a consistent systemic accumulation of Acylcarnitines (Figures 4e and 6d), a clinical marker of incomplete beta‐oxidation and mitochondrial stress.

Finally, the loss of Biliverdin in serum (Figure 4d) highlighted a specific deficit in endogenous antioxidant capacity. Together, these biomarkers, TMAO enrichment, Acylcarnitine accumulation, and Biliverdin/LPC depletion bridge our murine atlas with human translational endpoints (Li et al. 2025; Xiao et al. 2025; Zhou et al. 2024). A strategic strength of our study is the focus on aged (16‐month) mice, a time point reflecting mid‐life metabolic changes in humans (~45–50 years). By profiling this transition period, we uniquely captured the early metabolic vulnerabilities initiated by microbial decline before late‐life organ failure. While the redox stress observed here, manifesting as Biliverdin decline and TMAO elevation, may be considered moderate compared to end‐of‐life models, its early emergence confirms that the gut‐serum‐organ axis is an upstream driver of aging trajectories. This makes the identified TMAO‐LPC‐Acylcarnitine signature particularly valuable as a set of preventative, rather than purely diagnostic, biomarkers.

### 4.7 The IAA‐TMAO‐LPC Signature as a Modifiable Therapeutic Hub

The disruption of microbial metabolite containment emerged as a mechanistic centerpiece of gut‐liver‐brain inflammaging. While the Kynurenine pathway showed variable responses, the cortical accumulation of toxic indoles (Indole‐3‐acetic acid) and TMAO was a robust finding that explicitly suggested an age‐related compromise of blood–brain barrier selectivity (Figure 7d). These metabolites are known mediators of microglial activation, excitotoxicity, and mitochondrial dysfunction, providing a plausible explanation for the cortical neurotransmitter imbalance observed in aged mice (Brunt et al. 2021; Kurhaluk et al. 2025). Together, these findings implicate gut‐derived signal accumulation (IAA, TMAO) rather than IDO1 activation alone as a unifying mechanism driving neuroinflammation in the brain. This toxic accumulation phenotype in the cortex, coupled with the failure of hepatic lipid mobilization (LPC accumulation) and serum antioxidant depletion (Biliverdin), defines a multi‐hit model of metabolic aging. This IAA‐TMAO‐LPC signature represents a tractable therapeutic target; interventions capable of restoring gut barrier function or enhancing hepatic lipid transport could theoretically block this systemic propagation of redox stress (Ghosh et al. 2022; Xu et al. 2024).

### 4.8 Implications, Limitations, and Future Directions

This study was conducted exclusively in male C57BL/6 mice to minimize the confounding influence of sex‐specific hormonal and metabolic variations, which are known to affect gut microbiota composition, redox homeostasis, and lipid metabolism. This controlled design enhanced the clarity and interpretability of systemic aging trajectories across organs. While our meta‐analysis points toward causality, the atlas itself is associative and requires direct metabolite‐rescue experiments for confirmation. Future work should include female cohorts to capture sex‐specific trajectories and longitudinal sampling to assess the predictive value of identified biomarkers.

## 5 Conclusion

Our multi‐organ, multi‐omics atlas provides a detailed mechanistic framework demonstrating how early aging is characterized by gut microbial functional decline and mid‐life metabolic remodeling that propagates across the serum‐liver‐lung‐cortex axis. We identified a conserved molecular fingerprint of aging defined by the loss of protective circulating metabolites, specifically lysophosphatidylcholines (LPCs), and the concurrent accumulation of pro‐oxidative microbial catabolites, particularly trimethylamine N‐oxide (TMAO) and indole‐3‐acetic acid (IAA). This metabolic drift drives distinct tissue‐specific vulnerabilities, including hepatic lipid retention, pulmonary ferroptosis susceptibility, and cortical neurochemical dysregulation. Critically, our integrated meta‐analysis of 40 independent studies spanning natural aging models, fecal microbiota transplantation (FMT), and probiotic interventions provided quantitative evidence that this microbial remodeling causally drives systemic aging phenotypes. This analysis confirmed that gut microbiota‐targeted therapies effectively restore intestinal barrier integrity (e.g., upregulating ZO‐1 and MUC2), suppress systemic inflammatory factors (e.g., IL‐6 and TNF‐α), and mitigate distal oxidative stress (e.g., reducing MDA and restoring SOD and GSH). Together, these findings elevate the gut‐derived IAA‐TMAO‐LPC signature as a validated, modifiable mechanism of systemic aging, providing translationally relevant biomarkers and clear therapeutic targets for extending healthspan.

## Author Contributions

**Sanaullah Sajid:** writing – original draft, meta‐analysis and supervision. **Jieliang Huang:** methodology (animal experiments), data analysis. **Shaofang Kong:** data analysis and visualization. **Chengze Lai:** methodology. **Zhuoxin Tan:** meta‐analysis and data analysis. **Yiming Shao:** resources, funding acquisition. **Lianxian Guo:** funding acquisition, conceptualization, supervision, writing – review and editing.

## Funding

This work was supported by the National Natural Science Foundation of China, 82273757, 82574226. Natural Science Foundation of Guangdong Province, 2023B1515020106. Discipline Construction Project of Guangdong Medical University, 4SG25295G, 4SG25239G.

## Disclosure

Contact for Reagent and Resource Sharing: Further information and requests for resources and reagents should be directed to and will be fulfilled by the Lead Contact, Lianxian Guo (glx525@gdmu.edu.cn).

## Conflicts of Interest

The authors declare no conflicts of interest.

## Supporting information


**Data S1:** acel70433‐sup‐0001‐Supinfo.zip.

## Data Availability

The raw metabolomics and meta‐analysis datasets generated and analyzed in this study are publicly available in the Mendeley Data repository at https://data.mendeley.com with the identifier DOI: 10.17632/9ys25jktgw.2. All additional data supporting the findings of this study are available within the article and its Supporting Information files.
