# Supplementary material for: A Multi‐Organ Atlas Links Gut Microbial Metabolites to Systemic Redox Changes in Aging Mice
Source: Aging Cell. 2026 Mar 9;25(3):e70433. doi: 10.1111/acel.70433 (PMC12968584; doi:10.1111/acel.70433)
Supplement: Supplementary file 1 — Data S1: acel70433‐sup‐0001‐Supinfo.zip. [file ACEL-25-e70433-s001.zip › acel70433-sup-0001-Supinfo/Meta-Analysis Paper and Criteria.docx]

Table S1 Inclusion criteria for meta-analysis

| Number | Inclusion criteria |
| --- | --- |
| 1 | The literature was published from 2016 to January 2026. |
| 2 | Literature on establishing mouse models of natural aging, transplanting fecal microbiota from young mouse donors to aged mice, and supplementing probiotics to aged mice. |
| 3 | It contains inflammatory factors(IL-10、IL-6、IL-1β、TNF-α), barrier indicators(ZO-1、Cldn1、Ocld1、MUC2) , oxidative stress indicators(MDA、SOD、GSH), and also includes I-FABP, Endotoxin, PAI-1, p16, MCP-1. |
| 4 | Clarify the indicators of the detection area. |

Table S2 A literature list of meta-analyses on natural aging

| Search term | Database | Total |
| --- | --- | --- |
| (("gut microbiota" OR "microbiome" OR "fecal microbiota transplantation") AND ("aging" OR "longevity" OR "senescence")) AND ("inflammation" OR "C-reactive protein" OR ("curr res psychol" OR "crp") OR "interleukin-6" OR "IL-6" OR "TNF-alpha") | PubMed | 1175 |
| "probiotic" OR "Lactobacil" OR "Bifidobacter" OR "fecal microbiota transplant" OR "FMT" AND "aging" OR "ageing" OR "senescence" AND "IL-6" OR"IL-1β" OR "TNF-α"AND "ZO-1" OR "occludin" OR "claudin" AND "SOD" OR "MDA" OR "GSH" | Google Scholar | 1770 |

Table S3 A literature list of meta-analyses on natural aging

| **Number** | **Study** | **Aged** | | | **Young** | | | **Animal** | **Time** | **Treatment Strategies** | **Outcomes** | **Tissues** |
| --- | --- | --- | --- | --- | --- | --- | --- | --- | --- | --- | --- | --- |
|  |  | **Mean** | **SD** | **N** | **Mean** | **SD** | **N** |  |  |  |  |  |
| 1 | Aimée Parker（2022）^[1]^ | 1.34 | 0.31 | 7 | 1.08 | 0.16 | 8 | C57BL/6J male mice | 3 and 24 months old | young (3 months),aged (24 months) male mice | I-FABP | Serum |
|  |  | 52.10 | 63.33 | 5 | 24.65 | 1.37 | 5 |  |  |  | IL-6 | Serum |
|  |  | 1.03 | 0.22 | 6 | 0.73 | 0.35 | 6 |  |  |  | TNF-α | Intestine |
| 2 | Annette Brandt（2022）^[2]^ | 3.31 | 0.39 | 6 | 1.86 | 0.22 | 6 | C57BL/6J male mice(n = 5–9) | 3 and 24 months old | young (3 months),aged (24 months) male mice | PAI-1 | Plasma |
|  |  | 2571.00 | 878.00 | 6 | 100.00 | 9.60 | 6 |  |  |  | p16 | Liver |
|  |  | 0.41 | 0.08 | 5 | 0.19 | 0.04 | 5 |  |  |  | Endotoxin | Plasma |
| 3 | Dandan Wang（2025）^[3]^ | 0.56 | 0.07 | 3 | 1.02 | 0.12 | 3 | Female aged BalB/c mice | Two‐ and eighteen‐month‐old | the young group and the aging groups with and without GPN supplementation | MUC2 | Intestine |
|  |  | 0.97 | 0.09 | 3 | 0.97 | 0.09 | 3 |  |  |  | ZO-1 | Intestine |
|  |  | 0.77 | 0.06 | 3 | 0.99 | 0.11 | 3 |  |  |  | Ocln1 | Intestine |
|  |  | 0.14 | 0.04 | 9 | 0.14 | 0.04 | 9 |  |  |  | Endotoxin | Serum |
| 4 | Sidharth P Mishra（2024）^[4]^ | 0.98 | 0.31 | 10 | 3.04 | 1.18 | 9 | C57BL/6J (B6) male mice | 10-week (young donor, n = 10) and 78-week (aged donor, n = 10) | 10-week (young donor) and 78-week (aged donor) | Il-1β | Ileum |
|  |  | 1.01 | 0.27 | 10 | 3.84 | 1.22 | 10 |  |  |  | IL-6 | Ileum |
|  |  | 1.00 | 0.19 | 10 | 4.62 | 1.67 | 10 |  |  |  | TNF-α | Ileum |
|  |  | 0.98 | 0.37 | 10 | 3.67 | 2.03 | 10 |  |  |  | Il-1β | Colon |
|  |  | 1.05 | 0.45 | 10 | 4.60 | 2.09 | 10 |  |  |  | IL-6 | Colon |
|  |  | 1.04 | 0.42 | 10 | 5.04 | 2.17 | 10 |  |  |  | TNF-α | Colon |
|  |  | 32.91 | 10.41 | 10 | 156.45 | 31.93 | 10 |  |  |  | IL-6 | Serum |
|  |  | 61.41 | 44.45 | 10 | 255.37 | 104.30 | 10 |  |  |  | TNF-α | Serum |
| 5 | Kyung-Ah Kim（2016）^[5]^ | 3.40 | 0.99 | 8 | 2.33 | 0.60 | 8 | C57BL/6J male mice | 4 or 18 months old | 4 or 18 months old | Endotoxin | Fecal |
|  |  | 9.34 | 4.84 | 8 | 5.27 | 1.84 | 8 |  |  |  | Endotoxin | Plasma |
|  |  | 0.12 | 0.13 | 8 | 0.03 | 0.08 | 8 |  |  |  | p16 | Colon |
| 6 | Lu Wang（2024）^[6]^ | 2.91 | 0.75 | 5 | 0.30 | 0.09 | 5 | male Sprague-Dawley rats | male Sprague-Dawley rats aged 24 months and male Sprague-Dawley rats aged 8 weeks | a young control group, an aged control group | p16 | Lung |
|  |  | 3.48 | 1.47 | 5 | 0.38 | 0.16 | 5 |  |  |  | p16 | Intestine |
|  |  | 602.41 | 301.20 | 5 | 1506.02 | 240.96 | 5 |  |  |  | SOD | Lung |
|  |  | 7048.19 | 1265.06 | 5 | 17771.08 | 2048.19 | 5 |  |  |  | SOD | Intestine |
|  |  | 23.04 | 4.88 | 5 | 12.47 | 2.17 | 5 |  |  |  | MDA | Lung |
|  |  | 20.60 | 3.79 | 5 | 11.65 | 1.90 | 5 |  |  |  | MDA | Intestine |
|  |  | 3.60 | 0.86 | 5 | 2.85 | 0.56 | 5 |  |  |  | GSH | Lung |
|  |  | 1.83 | 0.22 | 5 | 1.61 | 0.24 | 5 |  |  |  | GSH | Intestine |
|  |  | 2948.57 | 262.86 | 5 | 925.71 | 80.00 | 5 |  |  |  | IL-1β | Lung |
|  |  | 1451.43 | 182.86 | 5 | 1302.86 | 125.71 | 5 |  |  |  | IL-1β | Intestine |
|  |  | 2215.91 | 409.09 | 5 | 1045.45 | 170.45 | 5 |  |  |  | IL-6 | Lung |
|  |  | 2318.18 | 386.36 | 5 | 1840.91 | 170.45 | 5 |  |  |  | IL-6 | Intestine |
|  |  | 11.01 | 3.21 | 5 | 6.19 | 2.06 | 5 |  |  |  | IL-1β | Serum |
|  |  | 72.94 | 5.05 | 5 | 40.60 | 7.57 | 5 |  |  |  | IL-6 | Serum |
|  |  | 3.33 | 0.83 | 5 | 0.43 | 0.37 | 5 |  |  |  | p16 | Brain |
|  |  | 1339.85 | 400.98 | 5 | 2581.91 | 322.74 | 5 |  |  |  | SOD | Brain |
|  |  | 181.87 | 12.17 | 5 | 123.48 | 22.51 | 5 |  |  |  | MDA | Brain |
|  |  | 2.18 | 0.28 | 5 | 2.36 | 0.36 | 5 |  |  |  | GSH | Brain |
|  |  | 1333.33 | 211.38 | 5 | 1081.30 | 308.94 | 5 |  |  |  | IL-1β | Brain |
|  |  | 990.85 | 79.27 | 5 | 881.10 | 131.10 | 5 |  |  |  | IL-6 | Brain |
|  |  | 5.58 | 1.07 | 5 | 0.95 | 0.31 | 5 |  |  |  | p16 | Liver |
|  |  | 730.09 | 82.96 | 5 | 1188.05 | 129.42 | 5 |  |  |  | SOD | Liver |
|  |  | 22.57 | 2.72 | 5 | 15.73 | 2.26 | 5 |  |  |  | MDA | Liver |
|  |  | 3.70 | 0.54 | 5 | 3.34 | 0.72 | 5 |  |  |  | GSH | Liver |
|  |  | 138.46 | 28.52 | 5 | 59.29 | 13.88 | 5 |  |  |  | IL-1β | Liver |
|  |  | 70.49 | 15.04 | 5 | 49.44 | 11.84 | 5 |  |  |  | IL-6 | Liver |
| 7 | Sarah E Webster（2023）^[7]^ | 2.58 | 0.79 | 14 | 3.51 | 1.14 | 16 | BALB/c-ByJ mice | either 3-months or 18–26-months of age | aged to either 3-months or 18–26-months of age in one of two separate animal facilities | IL-1β | Serum |
|  |  | 25.39 | 3.56 | 14 | 6.79 | 2.92 | 16 |  |  |  | IL-6 | Serum |
|  |  | 27.47 | 4.09 | 14 | 15.85 | 4.28 | 16 |  |  |  | IL-10 | Serum |
| 8 | Paola Elizabeth Gámez-Macías（2024）^[8]^ | 0.42 | 0.11 | 10 | 0.95 | 0.16 | 10 | C57BL/6J female mice | 2 months old (n = 10), and 18 months old (n = 10) | 2 months old, and 18 months old | ZO-1 | Intestine |
|  |  | 2.96 | 0.48 | 10 | 0.93 | 0.21 | 10 |  |  |  | TNF-α | Colon |
|  |  | 0.86 | 0.12 | 10 | 0.96 | 0.10 | 10 |  |  |  | IL-6 | Colon |
|  |  | 0.36 | 0.12 | 10 | 0.91 | 0.14 | 10 |  |  |  | IL-10 | Colon |
|  |  | 2.58 | 0.38 | 10 | 0.96 | 0.17 | 10 |  |  |  | MCP-1 | Colon |
| 9 | Shaohua Chen（2024）^[9]^ | 0.58 | 0.09 | 4 | 1.00 | 0.13 | 4 | C57BL/6J mice | young (2–3 months) and old (20–22 months) mice | young (2–3 months) and old (20–22 months) mice | ZO-1 | Intestine |
| 10 | Yanli Li（2020）^[10]^ | 93.68 | 24.62 | 6 | 26.32 | 3.08 | 6 | male SD rats | Young (~ 3 months) and aged (20 ~ 24 months) male SD rats | Young (~ 3 months) and aged (20 ~ 24 months) male SD rats | IL-1β | Serum |
|  |  | 53.25 | 9.97 | 6 | 26.52 | 4.59 | 6 |  |  |  | TNF-α | Serum |
|  |  | 56.80 | 8.00 | 6 | 39.40 | 5.00 | 6 |  |  |  | IL-6 | Serum |
|  |  | 29.34 | 6.34 | 6 | 52.92 | 5.90 | 6 |  |  |  | SOD | Serum |
|  |  | 87.65 | 4.94 | 6 | 59.01 | 5.93 | 6 |  |  |  | MDA | Serum |
| 11 | Yuji Morita（2018）^[11]^ | 359.79 | 72.75 | 6 | 99.21 | 15.87 | 5 | C57BL/6N female Mice | Young (1-month-old, n = 5), or aged (16-months-old, n = 12 in each group) | Young (1-month-old), or aged (16-months-old) | MCP-1 | Serum |
|  |  | 568.73 | 210.24 | 6 | 118.60 | 13.48 | 5 |  |  |  | IL-1β | Serum |
|  |  | 77.97 | 38.79 | 6 | 9.10 | 1.58 | 5 |  |  |  | IL-6 | Serum |
|  |  | 738.22 | 302.36 | 6 | 109.95 | 19.63 | 5 |  |  |  | TNF-α | Serum |
| 12 | Ravichandra Vemuri（2019）^[12]^ | 200.85 | 12.82 | 8 | 231.48 | 2.85 | 8 | C5BL/6J mice | young mice 3–4 weeks old (n = 16) and aging mice (n = 16) 35–36 weeks old | young mice 3–4 weeks old and aging mice 35–36 weeks old | TNF-α | Serum |
|  |  | 132.56 | 4.07 | 8 | 130.23 | 11.63 | 8 |  |  |  | IL-10 | Serum |
|  |  | 53.67 | 7.63 | 8 | 75.99 | 5.37 | 8 |  |  |  | IL-1β | Serum |
|  |  | 1277.78 | 55.56 | 8 | 1138.89 | 41.67 | 8 |  |  |  | MCP-1 | Serum |
| 13 | Melissa N Conley（2016）^[13]^ | 62.93 | 56.70 | 5 | 7.84 | 6.23 | 5 | C57Bl/6 female mice | Young (2 mo.) and aged (26 mo.) female C57Bl/6 mice | Young (2 mo.) and aged (26 mo.) female C57Bl/6 mice | MCP-1 | Serum |
| 14 | Yingli Jing（2024）^[14]^ | 26.48 | 10.81 | 6 | 10.56 | 8.33 | 6 | C57BL/6 J female mice | young (3 month old) and aged (22 month old) female C57BL/6 J mice | young (3 month old) and aged (22 month old) female C57BL/6 J mice | IL-1β | Colon |
|  |  | 18.01 | 10.22 | 6 | 3.69 | 4.14 | 6 |  |  |  | TNF-α | Colon |
|  |  | 82.75 | 37.24 | 6 | 43.90 | 16.03 | 6 |  |  |  | MCP-1 | Colon |
|  |  | 129.74 | 82.39 | 7 | 167.83 | 104.44 | 6 |  |  |  | IL-10 | Colon |
|  |  | 0.53 | 0.20 | 4 | 0.26 | 0.06 | 4 |  |  |  | IL-1β | Brain |
|  |  | 0.44 | 0.17 | 4 | 0.18 | 0.11 | 4 |  |  |  | TNF-α | Brain |
| 15 | Vienna E Brunt（2021）^[15]^ | 5.15 | 0.37 | 14 | 3.69 | 0.42 | 9 | C57BL/6N male mice | 8 weeks of age (N = 34) and 20–24 months of age (N = 16) | 8 weeks of age and 20–24 months of age | IL-1β | Brain |
|  |  | 14.90 | 1.39 | 14 | 10.26 | 1.97 | 9 |  |  |  | IL-6 | Brain |
|  |  | 4.00 | 0.74 | 14 | 1.62 | 0.22 | 9 |  |  |  | TNF-α | Brain |
|  |  | 23.67 | 2.60 | 14 | 16.04 | 2.69 | 9 |  |  |  | IL-10 | Brain |
| 16 | Renjie Shi（2024）^[16]^ | 226.02 | 65.39 | 6 | 73.17 | 13.58 | 6 | C57BL/6J male mice | the aging group (n = 20) and 2-month-old male C57BL/6J mice (Young group,n = 10) | the aging group and 2-month-old male C57BL/6J mice | TNF-α | Serum |
|  |  | 26.89 | 6.35 | 6 | 8.03 | 2.88 | 6 |  |  |  | IL-1β | Serum |
|  |  | 99.85 | 33.19 | 6 | 229.75 | 49.67 | 6 |  |  |  | SOD | Serum |
|  |  | 11.93 | 3.84 | 6 | 3.56 | 1.10 | 6 |  |  |  | MDA | Serum |
|  |  | 1.57 | 0.31 | 6 | 0.99 | 0.21 | 6 |  |  |  | TNF-α | Brain |
|  |  | 1.60 | 0.71 | 6 | 1.00 | 0.23 | 6 |  |  |  | IL-1β | Brain |
|  |  | 0.63 | 0.07 | 6 | 1.01 | 0.23 | 6 |  |  |  | SOD | Brain |
|  |  | 0.38 | 0.11 | 6 | 1.00 | 0.07 | 6 |  |  |  | ZO-1 | Colon |
|  |  | 0.43 | 0.07 | 6 | 1.04 | 0.34 | 6 |  |  |  | Cldn1 | Colon |
|  |  | 0.54 | 0.12 | 6 | 1.00 | 0.14 | 6 |  |  |  | Ocln1 | Colon |
| 17 | Jiali Ni（2025）^[17]^ | 4.83 | 1.62 | 8 | 4.46 | 0.89 | 8 | C57BL/6 male mice | Young (8-10 weeks) and aging (17–18 months) | Young (8-10 weeks) and aging (17–18 months) | IL-1β | Serum |
|  |  | 3.68 | 2.13 | 8 | 2.86 | 0.99 | 8 |  |  |  | IL-6 | Serum |
|  |  | 20.31 | 9.58 | 8 | 23.63 | 2.98 | 8 |  |  |  | TNF-α | Serum |
|  |  | 1.05 | 0.31 | 8 | 1.16 | 0.33 | 8 |  |  |  | MUC2 | Colon |
|  |  | 1.13 | 0.15 | 8 | 1.05 | 0.35 | 8 |  |  |  | ZO-1 | Colon |
| 18 | Nicholas A Crossland（2023）^[18]^ | 116.20 | 9.78 | 8 | 77.60 | 1.28 | 8 | C57BL6/J mice | Young (6 wk. N = 10 male + 10 female) and old (72 wk. N = 10 male + 10 female) | Young (6 wk.) and old (72 wk.) | IL-6 | Serum |
|  |  | 102.00 | 5.00 | 8 | 77.60 | 2.50 | 8 |  |  |  | IL-6 | Colon |
|  |  | 162.30 | 4.40 | 8 | 132.40 | 3.00 | 8 |  |  |  | IL-1β | Colon |
|  |  | 184.60 | 7.40 | 8 | 142.20 | 8.90 | 8 |  |  |  | TNF-α | Colon |

Table S4 A list of literature on meta-analysis of fecal microbiota transplantation

| **Number** | **Study** | **Aged+Young FMT** | | | **Aged OR Aged+Aged FMT** | | | **Animal** | **Time** | **Treatment Strategies** | **Outcomes** | **Tissues** |
| --- | --- | --- | --- | --- | --- | --- | --- | --- | --- | --- | --- | --- |
|  |  | **Mean** | **SD** | **N** | **Mean** | **SD** | **N** |  |  |  |  |  |
| 1 | Xiangjun Zeng（2023）^[19]^ | 1.02 | 0.29 | 6 | 2.24 | 0.40 | 6 | C57BL/6 mice | Young (7-8 weeks) and aged (20-24 months) | the gut microbiota from healthy young donor mice or aged donor mice were transplanted into aged recipient mice (FMT-YA and FMT-AA group, respectively) | IL-6 | Intestine |
| 2 | Aimée Parker（2022）^[1]^ | 0.96 | 0.17 | 6 | 1.34 | 0.31 | 7 | C57BL/6J Male mice | Male C57BL/6J mice aged 3, 24 months | aged mice receiving young donor microbiota | I-FABP | Serum |
|  |  | 38.72 | 21.96 | 5 | 52.10 | 63.33 | 5 |  |  |  | IL-6 | Serum |
|  |  | 0.64 | 0.10 | 8 | 1.03 | 0.22 | 6 |  |  |  | TNF-a | Intestine |
| 3 | Annette Brandt（2022）^[2]^ | 1.60 | 0.20 | 7 | 1.50 | 0.10 | 7 | C57BL/6J male mice | 3 months old male C57BL/6J mice and 17 months old male C57BL/6J mice | Mice were then randomly assigned to two groups treated with fecal microbiota from 1) young (o + yFMT) or 2) from old mice (o + oFMT) | PAI-1 | Plasma |
|  |  | 99.50 | 28.64 | 7 | 60.68 | 12.44 | 7 |  |  |  | Ocln1 | Intestine |
|  |  | 99.86 | 7.40 | 7 | 126.16 | 18.08 | 7 |  |  |  | p16 | Liver |
|  |  | 99.50 | 18.84 | 7 | 66.71 | 9.05 | 7 |  |  |  | ZO-1 | Intestine |
|  |  | 0.24 | 0.02 | 7 | 0.26 | 0.01 | 7 |  |  |  | Endotoxin | Plasma |
|  |  | 100.00 | 13.00 | 7 | 89.10 | 9.70 | 7 |  |  |  | Muc2 | Intestine |
| 4 | Dandan Wang（2025）^[3]^ | 0.91 | 0.16 | 6 | 0.61 | 0.11 | 6 | BALB/c mice | Two‐ and eighteen‐month‐old BALB/c mice | the YA control group (aged mice that accepted microbiota from young donors)；the AA control group (aged mice that accepted microbiota from aged donors) | ZO-1 | Intestine |
|  |  | 1.03 | 0.09 | 6 | 0.55 | 0.21 | 6 |  |  |  | Ocln1 | Intestine |
|  |  | 0.86 | 0.07 | 6 | 0.56 | 0.08 | 6 |  |  |  | MUC2 | Intestine |
| 5 | Sidharth P Mishra（2024）^[4]^ | 0.26 | 0.16 | 5 | 1.06 | 0.64 | 5 | C57BL/6J (B6) male mice | 10-week (young donor, n = 10) and 78-week (aged donor, n = 10) | We then transplanted young donor microbiota into 9 mice and old donor microbiota into 9 mice | IL-1b | Ileum |
|  |  | 0.28 | 0.15 | 5 | 1.01 | 0.57 | 5 |  |  |  | IL-6 | Ileum |
|  |  | 0.31 | 0.28 | 5 | 1.09 | 0.87 | 5 |  |  |  | TNF-a | Ileum |
|  |  | 0.30 | 0.24 | 5 | 0.99 | 0.45 | 5 |  |  |  | IL-1b | Brain |
|  |  | 0.32 | 0.29 | 5 | 1.05 | 0.37 | 5 |  |  |  | IL-6 | Brain |
|  |  | 0.40 | 0.27 | 5 | 0.95 | 0.40 | 5 |  |  |  | TNF-a | Brain |
|  |  | 1.00 | 0.13 | 10 | 0.07 | 0.05 | 10 |  |  |  | MUC2 | Ileum |
|  |  | 1.04 | 0.43 | 10 | 0.36 | 0.33 | 10 |  |  |  | MUC2 | Colon |
| 6 | Chak Kwong Cheng（2024）^[20]^ | 67.68 | 10.10 | 8 | 78.17 | 11.16 | 8 | C57BL/6 male mice | (young: 8 weeks old (n = 8); aged: >75 weeks old (n = 16) | aged and middle-aged recipient mice were given via oral gavage 150 μL of microbiota suspension from young donors C57BL/6 mice | IL-6 | Serum |
|  |  | 20.87 | 5.39 | 8 | 24.89 | 4.96 | 8 |  |  |  | TNF-a | Serum |
|  |  | 3.38 | 0.86 | 8 | 4.20 | 0.68 | 8 |  |  |  | Endotoxin | Fecal |
|  |  | 20.75 | 2.89 | 8 | 24.94 | 4.78 | 8 |  |  |  | Endotoxin | Serum |
|  |  | 2.01 | 0.27 | 8 | 2.31 | 0.34 | 8 |  |  |  | I-FABP | Serum |
| 7 | Nicholas A Crossland（2023）^[18]^ | 28.29 | 7.00 | 19 | 39.92 | 8.25 | 18 | A/J mice | Eight-week-old A/J mice (N = 20 (10 male + 10 female) per group) | RY, Recipients of FMT from Young mice; RO, Recipients of FMT from Old mice | IL-6 | Colon |
|  |  | 32.00 | 5.67 | 19 | 38.81 | 8.35 | 18 |  |  |  | IL-1b | Colon |
|  |  | 26.32 | 2.38 | 19 | 34.97 | 4.58 | 18 |  |  |  | TNF-a | Colon |
| 8 | Xiuxiu Chen（2024）^[21]^ | 1.20 | 0.05 | 6 | 0.95 | 0.10 | 6 | C57BL/6 male mice | aged 18 months and young 8-week-old | FMT involved transferring fecal samples from young mice (8 weeks old) to older mice (18 months old) | MUC2 | Ileum |
|  |  | 1.35 | 0.10 | 6 | 1.37 | 0.18 | 6 |  |  |  | Cldn1 | Ileum |
|  |  | 1.19 | 0.04 | 6 | 1.17 | 0.06 | 6 |  |  |  | Ocln1 | Ileum |
|  |  | 0.97 | 0.18 | 6 | 1.03 | 0.11 | 6 |  |  |  | ZO-1 | Ileum |
|  |  | 0.85 | 0.10 | 6 | 1.04 | 0.08 | 6 |  |  |  | IL-1b | Ileum |
|  |  | 0.90 | 0.13 | 6 | 1.29 | 0.14 | 6 |  |  |  | IL-6 | Ileum |
|  |  | 0.90 | 0.07 | 6 | 1.05 | 0.10 | 6 |  |  |  | TNF-a | Ileum |
|  |  | 1.07 | 0.10 | 6 | 0.96 | 0.10 | 6 |  |  |  | IL-10 | Ileum |
|  |  | 1.70 | 0.21 | 6 | 1.84 | 0.23 | 6 |  |  |  | IL-1b | Serum |
|  |  | 7.52 | 0.43 | 6 | 7.45 | 0.12 | 6 |  |  |  | IL-6 | Serum |
|  |  | 5.87 | 0.23 | 6 | 6.89 | 0.33 | 6 |  |  |  | TNF-a | Serum |
|  |  | 14.45 | 1.59 | 6 | 9.88 | 1.34 | 6 |  |  |  | IL-10 | Serum |
|  |  | 0.43 | 0.03 | 6 | 0.43 | 0.05 | 6 |  |  |  | SOD | Intestine |
|  |  | 58.61 | 3.64 | 6 | 61.92 | 2.65 | 6 |  |  |  | MDA | Intestine |
| 9 | Juneyoung Lee（2020）^[22]^ | 0.91 | 0.34 | 9 | 0.82 | 0.38 | 7 | C57BL/6-GF male mice | Fresh fecal samples were collected at 9–10 am from wild-type young (2–3 months) or aged (18–20 months) donor male mice (N = 5–10 per group) | Fecal transplant gavages (FTGs) from aged (18–20 months) or young (2–3 months) male C57BL/6 mice into germ-free male C57BL/6 mice | IL-6 | Plasma |
|  |  | 7.84 | 3.47 | 9 | 5.66 | 2.68 | 9 |  |  |  | IL-10 | Plasma |
|  |  | 43.84 | 21.83 | 10 | 37.25 | 27.99 | 9 |  |  |  | MCP-1 | Plasma |
|  |  | 10.48 | 3.22 | 10 | 8.89 | 4.31 | 9 |  |  |  | TNF-a | Plasma |

Table S5 A list of literature on meta-analysis of probiotics

| **Number** | **Study** | **Aged+probiotics** | | | **Aged** | | | **Animal** | **Time** | **Treatment Strategies** | **Outcomes** | **Tissues** |
| --- | --- | --- | --- | --- | --- | --- | --- | --- | --- | --- | --- | --- |
|  |  | **Mean** | **SD** | **N** | **Mean** | **SD** | **N** |  |  |  |  |  |
| 1 | Xueqin Yang（2019）^[23]^ | 52.72 | 3.74 | 6 | 27.55 | 1.70 | 6 | 9-month-old SAMP8 Male mice | SAMP8 mice were divided into two groups (n = 12/group) using the grading score system and Y-maze test before the experiment, and received vehicle (water) or ProBiotic-4 (2 × 109 CFU) once daily for 12 weeks. | ProBiotic-4, a probiotic preparation composed of B. lactis (50%), L. casei (25%), B. bifidum (12.5%), and L. acidophilus (12.5%) | Cldn1 | Intestine |
|  |  | 75.84 | 6.04 | 6 | 23.15 | 2.35 | 6 |  |  |  | Ocln1 | Intestine |
|  |  | 37.58 | 2.71 | 6 | 9.14 | 0.68 | 6 |  |  |  | ZO-1 | Intestine |
|  |  | 8.21 | 1.17 | 6 | 26.45 | 3.95 | 6 |  |  |  | IL-6 | Intestine |
|  |  | 8.85 | 2.45 | 6 | 15.68 | 0.96 | 6 |  |  |  | TNF-α | Intestine |
| 2 | Ibrahim Yusufu（2021）^[24]^ | 33.17 | 36.96 | 9 | 5.64 | 2.03 | 9 | C57BL/6 male mice | Twenty-month-old male C57BL/6 mice | The animals were fed either standard TRP (0.2%), high-TRP (1.25%) diets for eight weeks. | IL-6 | Serum |
| 3 | Rui-Ding Li（2023）^[25]^ | 2.96 | 0.48 | 24 | 3.62 | 0.53 | 12 | Twenty-month-old C57BL/6J naturally aged mice (SPF) | Lactobacillus fermentum LTP1332 and Bacteroides fragilis LTBF12 | control (C) mice were gavaged with sterile saline (same volume as used in the probiotic combination) daily; the low-dose (L) group was gavaged with 1 × 107 CFU/mL Lactobacillus fermentum LTP1332 + 1 × 107 CFU/mL Bacteroides fragilis LTBF12 daily; the high-dose (H) group was gavaged with 1 × 109 CFU/mL Lactobacillus fermentum LTP1332 + 1 × 109 CFU/mL Bacteroides fragilis LTBF12 daily | MDA | Brain |
|  |  | 97.47 | 3.56 | 24 | 85.88 | 2.16 | 12 |  |  |  | SOD | Brain |
|  |  | 39.02 | 0.82 | 24 | 48.89 | 2.88 | 12 |  |  |  | IL-6 | Brain |
| 4 | Kaikwa Wuttisa（2025）^[26]^ | 56.00 | 2.57 | 4 | 66.57 | 5.71 | 4 | C56BL/6 male mice | Probiotic strains, Lactobacillus paracasei MSMC39-1 and Bifidobacterium animalis MSMC83 | old control (OC) group and aged group (AP) mice were supplemented with L. paracasei MSMC39-1 and B. animalis MSMC83 | TNF-α | Colon |
|  |  | 2.26 | 1.08 | 4 | 6.45 | 1.72 | 4 |  |  |  | IL-1β | Colon |
|  |  | 271.26 | 29.12 | 5 | 162.45 | 26.05 | 5 |  |  |  | SOD | Liver |
|  |  | 14.90 | 1.98 | 5 | 25.10 | 4.87 | 5 |  |  |  | MDA | Liver |
|  |  | 6.81 | 1.49 | 5 | 11.28 | 2.81 | 5 |  |  |  | TNF-α | Liver |
|  |  | 0.58 | 0.13 | 5 | 0.86 | 0.26 | 5 |  |  |  | IL-1β | Liver |
|  |  | 293.89 | 22.90 | 3 | 118.32 | 17.18 | 3 |  |  |  | SOD | Brain |
|  |  | 8.27 | 2.00 | 3 | 20.06 | 3.04 | 3 |  |  |  | MDA | Brain |
|  |  | 0.82 | 0.20 | 3 | 1.94 | 0.37 | 3 |  |  |  | TNF-α | Brain |
|  |  | 0.65 | 0.07 | 3 | 0.73 | 0.11 | 3 |  |  |  | IL-1β | Brain |
| 5 | Yihui Cai（2024）[27] | 3.58 | 0.89 | 10 | 5.30 | 1.21 | 10 | 16 week-old specific pathogen-free male SAMP8 mice | Lacticaseibacillus paracasei LC86 (deposit number: CGMCC No. 1.12731) | TThe control group (CTL), serving as the model control with 10 SAMP8 mice, received 0.2 mL of sterile water as a vehicle treatment daily;the intervention group, which also consisted of 10 SAMP8 mice and received treatment with L. paracasei LC86 (LC86), was administered a daily volume of 0.2 mL sterile water containing 1 × 109 CFU of LC86 | TNF-α | Serum |
|  |  | 7.64 | 1.81 | 10 | 9.03 | 1.66 | 10 |  |  |  | IL-6 | Serum |
|  |  | 159.85 | 31.53 | 10 | 184.73 | 35.95 | 10 |  |  |  | MCP-1 | Serum |
|  |  | 53.39 | 10.18 | 10 | 42.32 | 8.75 | 10 |  |  |  | IL-10 | Serum |
|  |  | 31.43 | 7.59 | 10 | 20.89 | 4.73 | 10 |  |  |  | SOD | Liver |
|  |  | 74.67 | 12.66 | 10 | 60.70 | 8.30 | 10 |  |  |  | GSH | Liver |
| 6 | Yuji Morita（2018）[11] | 244.71 | 17.20 | 6 | 359.79 | 72.75 | 6 | C57BL/6N female mice | Young (1-month-old, n = 5), or aged (16-months-old, n = 12 in each group) mice | The control group mice were fed AIN93Mand the Lactobacillus paracasei KW3110-fed mice (hereafter called the KW3110 group mice) were fed AIN93M containing 1 mg heat-killed L. paracasei KW3110/day/mouse for 6 months | MCP-1 | Serum |
|  |  | 210.24 | 29.65 | 6 | 568.73 | 210.24 | 6 |  |  |  | IL-1β | Serum |
|  |  | 12.66 | 4.75 | 6 | 77.97 | 38.79 | 6 |  |  |  | IL-6 | Serum |
|  |  | 286.65 | 51.05 | 6 | 738.22 | 302.36 | 6 |  |  |  | TNF-α | Serum |
| 7 | Sheng-Yao Wang（2025）[28] | 17.90 | 3.48 | 9 | 17.49 | 2.98 | 9 | BALB/c male mice | D-galactose-induced aging was modeled via the subcutaneous injection of D-galactose (100 mg/kg/day) for 8 weeks | D-gal control；D-gal + Lactobacillus kefiranofaciens HL1 | MDA | Serum |
|  |  | 19.09 | 3.55 | 9 | 17.60 | 4.21 | 9 |  |  |  | SOD | Serum |
|  |  | 1.58 | 0.42 | 9 | 1.81 | 0.51 | 9 |  |  |  | MDA | Liver |
|  |  | 25.32 | 4.87 | 9 | 24.35 | 7.14 | 9 |  |  |  | SOD | Liver |
|  |  | 10.00 | 2.38 | 9 | 13.86 | 2.48 | 9 |  |  |  | MDA | Brain |
|  |  | 9.63 | 1.27 | 9 | 5.91 | 1.71 | 9 |  |  |  | SOD | Brain |
|  |  | 1.25 | 0.72 | 9 | 2.15 | 0.97 | 9 |  |  |  | TNF-α | Serum |
|  |  | 1329.37 | 462.96 | 9 | 1818.78 | 350.53 | 9 |  |  |  | IL-1β | Serum |
|  |  | 14.41 | 5.22 | 9 | 21.57 | 4.89 | 9 |  |  |  | TNF-α | Liver |
|  |  | 8777.47 | 5439.56 | 9 | 9148.35 | 2307.69 | 9 |  |  |  | IL-1β | Liver |
|  |  | 11.84 | 3.05 | 9 | 17.37 | 4.74 | 9 |  |  |  | TNF-α | Brain |
|  |  | 39.55 | 19.93 | 9 | 44.31 | 14.28 | 9 |  |  |  | IL-1β | Brain |
| 8 | Sheng-Yao Wang（2025）^[28]^ | 15.66 | 4.14 | 9 | 17.57 | 2.90 | 9 | BALB/c male mice | D-galactose-induced aging was modeled via the subcutaneous injection of D-galactose (100 mg/kg/day) for 8 weeks | D-gal control；D-gal + Lactococcus lactis subsp. cremoris APL015 | MDA | Serum |
|  |  | 17.93 | 2.81 | 9 | 17.69 | 4.13 | 9 |  |  |  | SOD | Serum |
|  |  | 1.80 | 0.29 | 9 | 1.80 | 0.52 | 9 |  |  |  | MDA | Liver |
|  |  | 24.86 | 5.27 | 9 | 24.46 | 7.03 | 9 |  |  |  | SOD | Liver |
|  |  | 13.10 | 3.54 | 9 | 14.09 | 2.23 | 9 |  |  |  | MDA | Brain |
|  |  | 8.64 | 1.53 | 9 | 5.93 | 1.69 | 9 |  |  |  | SOD | Brain |
|  |  | 1.76 | 0.51 | 9 | 2.16 | 0.95 | 9 |  |  |  | TNF-α | Serum |
|  |  | 1542.55 | 452.13 | 9 | 1821.81 | 339.10 | 9 |  |  |  | IL-1β | Serum |
|  |  | 14.28 | 5.41 | 9 | 21.63 | 4.82 | 9 |  |  |  | TNF-α | Liver |
|  |  | 7273.97 | 3369.86 | 9 | 9123.29 | 2342.47 | 9 |  |  |  | IL-1β | Liver |
|  |  | 15.20 | 1.72 | 9 | 17.39 | 4.70 | 9 |  |  |  | TNF-α | Brain |
|  |  | 42.39 | 8.10 | 9 | 44.26 | 14.34 | 9 |  |  |  | IL-1β | Brain |
| 9 | Jin-Ju Jeong（2015）^[29]^ | 0.22 | 0.01 | 10 | 0.08 | 0.01 | 10 | Male Fischer 344 rats (18 months-old) | Lactobacillus C29 | AR, aged rats;ARC, aged rats treated with C29(n = 10) | Cldn1 | Colon |
|  |  | 0.05 | 0.01 | 10 | 0.03 | 0.01 | 10 |  |  |  | Ocln1 | Colon |
|  |  | 0.07 | 0.00 | 10 | 0.03 | 0.00 | 10 |  |  |  | ZO-1 | Colon |
|  |  | 58.39 | 4.97 | 10 | 88.51 | 7.45 | 10 |  |  |  | TNF-α | Colon |
|  |  | 102.20 | 7.86 | 10 | 204.40 | 26.73 | 10 |  |  |  | IL-1β | Colon |
|  |  | 16.04 | 5.14 | 10 | 33.18 | 7.17 | 10 |  |  |  | IL-6 | Colon |
|  |  | 45.52 | 8.46 | 10 | 20.88 | 4.89 | 10 |  |  |  | IL-10 | Colon |
|  |  | 0.12 | 0.01 | 10 | 0.20 | 0.05 | 10 |  |  |  | p16 | Colon |
| 10 | Ravichandra Vemuri（2019）^[12]^ | 167.08 | 3.71 | 8 | 199.26 | 14.85 | 8 | C5BL/6J mice | aging mice (n = 16) 35–36 weeks old;L. acidophilus DDS-1 | aging control(ac) and aging probiotic(AP) | TNF-α | Serum |
|  |  | 158.59 | 9.09 | 8 | 133.33 | 4.04 | 8 |  |  |  | IL-10 | Serum |
|  |  | 32.35 | 3.92 | 8 | 52.94 | 7.84 | 8 |  |  |  | IL-1β | Serum |
|  |  | 859.15 | 133.80 | 8 | 1288.73 | 49.30 | 8 |  |  |  | MCP-1 | Serum |
| 11 | Minhong Ren（2022）^[30]^ | 104.19 | 5.81 | 6 | 83.55 | 4.52 | 6 | C57BL/6J male mice (SPF, 18 months old) | The patented Lactobacillus casei LTL1361 strain | control group (basal diet, administered saline) and LTL1361 group (basal diet, administered LTL1363) | SOD | Serum |
|  |  | 12.13 | 1.55 | 6 | 14.26 | 1.35 | 6 |  |  |  | MDA | Serum |
|  |  | 41.45 | 4.72 | 6 | 29.10 | 5.03 | 6 |  |  |  | SOD | Brain |
|  |  | 8.74 | 1.39 | 6 | 10.18 | 1.23 | 6 |  |  |  | MDA | Brain |
|  |  | 49.84 | 3.74 | 6 | 65.17 | 3.86 | 6 |  |  |  | TNF-α | Serum |
|  |  | 146.24 | 8.16 | 6 | 124.32 | 8.16 | 6 |  |  |  | IL-10 | Serum |
|  |  | 9.50 | 1.06 | 6 | 12.98 | 1.27 | 6 |  |  |  | TNF-α | Colon |
|  |  | 48.12 | 3.02 | 6 | 37.78 | 3.34 | 6 |  |  |  | IL-10 | Colon |
|  |  | 1.97 | 0.66 | 6 | 1.06 | 0.38 | 6 |  |  |  | ZO-1 | Intestine |
|  |  | 4.93 | 1.02 | 6 | 0.94 | 0.39 | 6 |  |  |  | Cldn1 | Intestine |
|  |  | 2.28 | 0.54 | 6 | 1.06 | 0.24 | 6 |  |  |  | ZO-1 | Colon |
|  |  | 1.87 | 0.57 | 6 | 1.10 | 0.54 | 6 |  |  |  | Cldn1 | Colon |
| 12 | Qingwei Zeng（2024）^[31]^ | 1.86 | 0.32 | 12 | 2.82 | 0.36 | 6 | Male BALB/c mice aged 6–8 weeks | B. pseudocatenulatum NCU-08 (CGMCC no. 26490) | Model (M) group (n = 8): administered intraperitoneal injections of 150 mg per kg per day D-gal (Sigma-Aldrich, G0750) and orally gavaged with normal saline at a volume equivalent to the probiotic.Low-dose probiotic(L) group (n = 8): administered intraperitoneal injections of 150 mg per kg per day D-gal and daily oral gavage of 1 × 107 CFU mL−1 B. pseudocatenulatum NCU-08. High-dose probiotic(H) group (n = 8): administered intraperitoneal injections of 150 mg per kg per day D-gal and daily oral gavage of 1 × 109 CFU mL−1 B. pseudocatenulatum NCU-08. | IL-1β | Brain |
|  |  | 1.47 | 0.27 | 12 | 2.30 | 0.35 | 6 |  |  |  | IL-6 | Brain |
|  |  | 1.58 | 0.29 | 12 | 2.21 | 0.22 | 6 |  |  |  | TNF-α | Brain |
|  |  | 0.63 | 0.11 | 6 | 0.30 | 0.11 | 3 |  |  |  | ZO-1 | Brain |
|  |  | 0.64 | 0.16 | 6 | 0.35 | 0.02 | 3 |  |  |  | Ocln1 | Brain |
|  |  | 6.84 | 0.68 | 12 | 8.28 | 0.44 | 6 |  |  |  | MDA | Brain |
|  |  | 255.47 | 18.67 | 12 | 217.49 | 9.42 | 6 |  |  |  | SOD | Brain |
|  |  | 11.81 | 1.46 | 12 | 14.84 | 0.90 | 6 |  |  |  | MDA | Serum |
|  |  | 55.01 | 4.62 | 12 | 42.89 | 5.28 | 6 |  |  |  | SOD | Serum |
|  |  | 1.84 | 0.45 | 12 | 2.43 | 0.22 | 6 |  |  |  | IL-1β | Colon |
|  |  | 1.44 | 0.28 | 12 | 2.18 | 0.41 | 6 |  |  |  | IL-6 | Colon |
|  |  | 1.38 | 0.20 | 12 | 1.91 | 0.17 | 6 |  |  |  | TNF-α | Colon |
|  |  | 0.45 | 0.15 | 6 | 0.15 | 0.05 | 3 |  |  |  | ZO-1 | Intestine |
|  |  | 0.64 | 0.16 | 6 | 0.38 | 0.02 | 3 |  |  |  | Ocln1 | Intestine |
| 13 | Renjie Shi（2024）^[16]^ | 124.03 | 25.90 | 6 | 226.04 | 65.42 | 6 | Two-month-old male C57BL/ 6J mice | L. plantarum LLY-606 | After 14 months of feeding, the mice were randomly divided into two groups: the aging group (n = 20) and the probiotic intervention group (aging+606 group, n = 20). LLY-606 (10∧9 CFU/ml) | TNF-α | Serum |
|  |  | 13.39 | 3.73 | 6 | 26.90 | 6.32 | 6 |  |  |  | IL-1β | Serum |
|  |  | 164.41 | 37.26 | 6 | 99.85 | 33.58 | 6 |  |  |  | SOD | Serum |
|  |  | 6.59 | 1.67 | 6 | 11.96 | 3.89 | 6 |  |  |  | MDA | Serum |
|  |  | 1.01 | 0.13 | 6 | 1.58 | 0.31 | 6 |  |  |  | TNF-α | Brain |
|  |  | 0.87 | 0.14 | 6 | 1.59 | 0.70 | 6 |  |  |  | IL-1β | Brain |
|  |  | 0.98 | 0.09 | 6 | 0.64 | 0.06 | 6 |  |  |  | SOD | Brain |
|  |  | 1.02 | 0.25 | 6 | 0.38 | 0.11 | 6 |  |  |  | ZO-1 | Colon |
|  |  | 1.04 | 0.14 | 6 | 0.43 | 0.07 | 6 |  |  |  | Cldn1 | Colon |
|  |  | 1.08 | 0.49 | 6 | 0.54 | 0.12 | 6 |  |  |  | Ocln1 | Colon |
| 14 | Sidharth P Mishra（2024）^[4]^ | 0.85 | 0.35 | 5 | 0.25 | 0.11 | 5 | mice of 10–12 and 78–82 weeks of age (conventional B6 gut-cleansed mice) | Butyrate | half of the old FMT recipients (n = 5) were administered butyrate (2%) in their drinking water for 2 weeks after the day of gut cleansing, and the other half drank normal water | MUC2 | Ileum |
|  |  | 1.06 | 0.58 | 5 | 0.33 | 0.33 | 5 |  |  |  | MUC2 | Colon |
|  |  | 1.09 | 0.60 | 5 | 2.78 | 1.50 | 5 |  |  |  | IL-1β | Colon |
|  |  | 1.19 | 0.33 | 5 | 2.82 | 1.47 | 5 |  |  |  | IL-6 | Colon |
|  |  | 1.34 | 0.60 | 5 | 2.64 | 1.07 | 5 |  |  |  | TNF-α | Colon |
|  |  | 1.29 | 0.80 | 5 | 2.87 | 1.23 | 5 |  |  |  | IL-1β | Brain |
|  |  | 1.09 | 0.52 | 5 | 3.11 | 2.12 | 5 |  |  |  | IL-6 | Brain |
|  |  | 0.62 | 0.41 | 5 | 3.15 | 1.57 | 5 |  |  |  | TNF-α | Brain |
| 15 | Xiaolin Liu（2025）^[32]^ | 136.73 | 4.37 | 6 | 104.86 | 2.71 | 6 | C57BL/6J male mice (6 wk) | Limosilactobacillus fermentum TD-3 and Lc. lactis MQ1-1 | D-gal groups were injected with 200 μL of sterile saline daily;The PM treatment group mice were intragastrically injected daily with 1.5 × 1010 cfu/kg BW mixture of Lb. fermentum TD-3 and Lc. lactis MQ1-1 (1:1) | SOD | Colon |
|  |  | 2.88 | 0.47 | 6 | 7.18 | 0.78 | 6 |  |  |  | MDA | Colon |
|  |  | 4.15 | 0.31 | 6 | 5.83 | 0.37 | 6 |  |  |  | IL-1β | Colon |
|  |  | 14.41 | 0.66 | 6 | 18.73 | 0.64 | 6 |  |  |  | IL-6 | Colon |
|  |  | 119.47 | 6.23 | 6 | 177.95 | 1.82 | 6 |  |  |  | TNF-α | Colon |
|  |  | 24.40 | 1.38 | 6 | 14.05 | 1.17 | 6 |  |  |  | IL-10 | Colon |
|  |  | 0.82 | 0.06 | 6 | 0.42 | 0.02 | 6 |  |  |  | Cldn1 | Colon |
|  |  | 1.39 | 0.06 | 6 | 0.30 | 0.02 | 6 |  |  |  | Ocln1 | Colon |
|  |  | 0.88 | 0.06 | 6 | 0.51 | 0.13 | 6 |  |  |  | ZO-1 | Colon |
| 16 | Porntipha Vitheejongjaroen（2022）^[33]^ | 109.76 | 5.49 | 5 | 70.61 | 5.12 | 5 | Eight-week-old male Sprague Dawley rats | Bifidobacterium animalis MSMC83 | The D-galactose group was treated with normal saline via oral gavage and subcutaneously injected with 5% (w/v) D-gal dissolved in normal saline (500 mg/kg body weight) once a day for 8 weeks. The D-gal plus probiotic group received the B. animalis MSMC83 by oral gavage at a concentration of 109 CFU/day and was injected daily with 5% (w/v) D-gal, as described earlier. | TNF-α | Liver |
|  |  | 12.37 | 1.63 | 5 | 4.53 | 1.38 | 5 |  |  |  | MDA | Plasma |
|  |  | 6.75 | 0.58 | 5 | 4.44 | 0.42 | 5 |  |  |  | MDA | Liver |
|  |  | 126.95 | 15.62 | 5 | 179.21 | 15.42 | 5 |  |  |  | SOD | Plasma |
|  |  | 115.34 | 11.35 | 5 | 172.63 | 9.67 | 5 |  |  |  | SOD | Liver |
| 17 | Huixian Han（2025）^[34]^ | 1.52 | 0.46 | 12 | 3.29 | 0.42 | 6 | Six-week-old male C57BL/6J mice | Lactiplantibacillus plantarum and Lacticaseibacillus casei | The control group received daily intraperitoneal and oral administrations of sterile saline. The other groups were administered D-galactose intraperitoneally (500 mg/kg·bw/day) and lychee fermentate orally at the indicated doses for eight consecutive weeks.The model group (n = 6), lychee fermentate group (n = 12) | p16 | Colon |
|  |  | 0.76 | 0.16 | 12 | 0.59 | 0.13 | 6 |  |  |  | MUC2 | Colon |
|  |  | 1.25 | 0.52 | 12 | 2.51 | 0.34 | 6 |  |  |  | IL-1β | Colon |
|  |  | 1.24 | 0.23 | 12 | 2.58 | 0.23 | 6 |  |  |  | IL-6 | Colon |
|  |  | 1.37 | 0.25 | 12 | 2.58 | 0.46 | 6 |  |  |  | TNF-α | Colon |
|  |  | 1.37 | 0.27 | 12 | 3.00 | 0.09 | 6 |  |  |  | MCP-1 | Colon |
|  |  | 24.52 | 5.08 | 12 | 31.33 | 3.72 | 6 |  |  |  | MDA | Serum |
|  |  | 5.26 | 0.70 | 12 | 3.61 | 0.74 | 6 |  |  |  | SOD | Serum |
| 18 | Yihong Zeng（2025）^[35]^ | 121.30 | 2.49 | 8 | 103.26 | 16.03 | 8 | ICR mice, 8 weeks, male | Bifidobacterium breve NKU BB 1–13 | The model group and B. breve group were administered daily injections of 300 mg/kg D galactose. Furthermore, B. breve group was administered 0.2 mL of NKU  BB 1–13 solution containing 1 × 109 CFU/mL on a daily basis, while the control and model groups received an equivalent volume of 0.9 % NaCl via oral administration. | SOD | Colon |
|  |  | 2.28 | 0.63 | 8 | 4.48 | 2.01 | 8 |  |  |  | MDA | Colon |
|  |  | 115.77 | 12.59 | 8 | 45.60 | 12.46 | 8 |  |  |  | SOD | Serum |
|  |  | 7.61 | 2.61 | 8 | 11.61 | 3.05 | 8 |  |  |  | MDA | Serum |
|  |  | 117.22 | 11.27 | 8 | 98.64 | 19.69 | 8 |  |  |  | GSH | Serum |
|  |  | 149.70 | 16.93 | 8 | 180.67 | 2.48 | 8 |  |  |  | TNF-α | Colon |
|  |  | 19.43 | 1.59 | 8 | 22.69 | 3.79 | 8 |  |  |  | IL-6 | Colon |
|  |  | 2.13 | 1.25 | 8 | 0.54 | 0.25 | 8 |  |  |  | MUC2 | Colon |
|  |  | 3.87 | 2.78 | 8 | 0.99 | 0.64 | 8 |  |  |  | Cldn1 | Colon |
| 19 | Jialong Fang（2025）^[36]^ | 0.81 | 0.28 | 10 | 2.13 | 0.36 | 10 | male C57BL/6J mice | CCFM8610 | Model group: mice were gavaged with 0.9% saline solution at 200 μL. CCFM8610 group: mice were gavaged with 200 μL of 5 × 109 CFU/mL of CCFM8610 | IL-1β | Colon |
|  |  | 1.20 | 0.09 | 10 | 3.07 | 0.52 | 10 |  |  |  | IL-6 | Colon |
|  |  | 1.37 | 0.24 | 10 | 0.42 | 0.12 | 10 |  |  |  | IL-10 | Colon |
|  |  | 1.53 | 0.16 | 10 | 2.66 | 0.39 | 10 |  |  |  | TNF-α | Colon |
|  |  | 1.62 | 0.13 | 10 | 2.19 | 0.42 | 10 |  |  |  | p16 | Colon |
|  |  | 631.16 | 61.14 | 10 | 347.14 | 35.50 | 10 |  |  |  | SOD | Colon |
|  |  | 1.56 | 0.14 | 10 | 2.54 | 0.16 | 10 |  |  |  | MDA | Colon |
|  |  | 644.37 | 19.37 | 10 | 573.94 | 14.08 | 10 |  |  |  | GSH | Colon |
|  |  | 1.65 | 0.18 | 10 | 0.51 | 0.08 | 10 |  |  |  | Ocln1 | Colon |
|  |  | 1.49 | 0.09 | 10 | 0.67 | 0.05 | 10 |  |  |  | ZO-1 | Colon |
|  |  | 3.14 | 0.39 | 10 | 0.79 | 0.08 | 10 |  |  |  | Cldn1 | Colon |
|  |  | 3.99 | 0.27 | 10 | 0.68 | 0.08 | 10 |  |  |  | MUC2 | Colon |
| 20 | Hui Nie（2024）^[37]^ | 327.05 | 12.16 | 5 | 238.30 | 24.32 | 5 | Seven-week-old male BALB/c mice | L. plantarum MWFLp-182 | The model group (LD) was intraperitoneally injected with 800 mg/kg of body weight of D-galactose (D-gal) every day and oral gavage with physiological saline. The treatment group (LH) was intraperitoneally injected with 800 mg/kg body weight of D-gal every day and oral gavage with 1 × 109 CFU/mL per mouse of L. plantarum MWFLp-182 every day | SOD | Serum |
|  |  | 4.21 | 0.22 | 5 | 5.38 | 0.11 | 5 |  |  |  | MDA | Serum |
|  |  | 216.01 | 14.35 | 5 | 160.12 | 9.82 | 5 |  |  |  | SOD | Brain |
|  |  | 2.79 | 0.46 | 5 | 5.19 | 1.30 | 5 |  |  |  | MDA | Brain |
|  |  | 7.83 | 0.16 | 5 | 17.65 | 0.28 | 5 |  |  |  | IL-1β | Serum |
|  |  | 20.65 | 0.87 | 5 | 9.82 | 0.46 | 5 |  |  |  | IL-10 | Serum |
|  |  | 15.46 | 0.65 | 5 | 36.16 | 1.80 | 5 |  |  |  | TNF-α | Serum |
|  |  | 27.73 | 0.66 | 5 | 38.72 | 1.22 | 5 |  |  |  | IL-1β | Liver |
|  |  | 65.99 | 2.56 | 5 | 50.17 | 0.60 | 5 |  |  |  | IL-10 | Liver |
|  |  | 74.53 | 3.56 | 5 | 89.33 | 3.00 | 5 |  |  |  | TNF-α | Liver |
|  |  | 33.14 | 0.72 | 5 | 52.49 | 1.75 | 5 |  |  |  | IL-1β | Colon |
|  |  | 65.40 | 1.38 | 5 | 45.44 | 3.86 | 5 |  |  |  | IL-10 | Colon |
|  |  | 71.99 | 1.20 | 5 | 94.67 | 3.61 | 5 |  |  |  | TNF-α | Colon |
|  |  | 0.78 | 0.09 | 5 | 0.48 | 0.11 | 5 |  |  |  | Ocln1 | Colon |
|  |  | 1.55 | 0.08 | 5 | 0.65 | 0.14 | 5 |  |  |  | Cldn1 | Colon |
|  |  | 1.07 | 0.10 | 5 | 0.61 | 0.06 | 5 |  |  |  | ZO-1 | Colon |
| 21 | Feng Chen（2023）^[38]^ | 1.60 | 0.19 | 8 | 2.23 | 0.20 | 8 | Eight-week-old male ICR mice | L. plantarum CCFM8661 | The L. plantarum CCFM8661 intervention group received 109 CFU of live bacteria by gavage daily,17,28 and the model groups received sterile saline by gavage (200 μL each) for 2 months. | p16 | Brain |
|  |  | 1.19 | 0.10 | 8 | 1.47 | 0.07 | 8 |  |  |  | TNF-α | Brain |
|  |  | 1.06 | 0.04 | 8 | 1.25 | 0.07 | 8 |  |  |  | IL-1β | Brain |
|  |  | 1.26 | 0.09 | 8 | 1.56 | 0.12 | 8 |  |  |  | IL-6 | Brain |
|  |  | 3.49 | 0.12 | 8 | 4.31 | 0.13 | 8 |  |  |  | MDA | Brain |
|  |  | 3.60 | 0.15 | 8 | 2.98 | 0.12 | 8 |  |  |  | GSH | Brain |
|  |  | 53.91 | 1.59 | 8 | 48.08 | 2.52 | 8 |  |  |  | SOD | Brain |
| 22 | Li-Han Chen（2021）^[39]^ | 4.62 | 0.27 | 4 | 6.00 | 0.24 | 4 | SAMP8 mice | LPPS23 | FA (aged mice administered saline), FPS23L (aged mice administered low dose 1 × 108 CFU LPPS23/mouse/day), and FPS23H (aged mice administered high dose 1 × 109 LPPS23 CFU/mouse/day) | TNF-α | Intestine |
|  |  | 280.66 | 17.69 | 4 | 382.08 | 4.72 | 4 |  |  |  | MCP-1 | Intestine |
| 23 | Lei Wu（2025）^[40]^ | 60.67 | 9.60 | 5 | 86.18 | 13.02 | 5 | SAMP8 (SPF grade) mice | Lactobacillus plantarum 124 (LP124) | MOD (SAMP8, 6 mice) groups received 0.2 mL of normal saline by gavage daily. For the LP124 test group (SAMP8, 6 mice), 0.2 mL of LP124 suspension containing 1.0 ± 0.05 × l09 colony forming units (CFU)/mL was administered | IL-1β | Colon |
|  |  | 363.86 | 26.84 | 5 | 460.40 | 40.33 | 5 |  |  |  | TNF-α | Colon |
|  |  | 227.56 | 25.67 | 5 | 163.75 | 17.14 | 5 |  |  |  | IL-10 | Colon |
|  |  | 65.20 | 8.60 | 5 | 94.05 | 7.77 | 5 |  |  |  | IL-6 | Colon |
|  |  | 47.44 | 6.66 | 5 | 33.67 | 3.01 | 5 |  |  |  | SOD | Colon |
|  |  | 10.93 | 0.56 | 5 | 15.33 | 0.75 | 5 |  |  |  | MDA | Colon |
|  |  | 124.50 | 13.94 | 5 | 87.36 | 5.17 | 5 |  |  |  | ZO-1 | Colon |
|  |  | 5.27 | 0.49 | 5 | 4.42 | 0.43 | 5 |  |  |  | Ocln1 | Colon |
|  |  | 583.09 | 57.63 | 5 | 411.87 | 29.77 | 5 |  |  |  | Cldn1 | Colon |
|  |  | 68.91 | 8.66 | 5 | 82.79 | 14.06 | 5 |  |  |  | IL-1β | Liver |
|  |  | 404.21 | 42.46 | 5 | 473.44 | 80.07 | 5 |  |  |  | TNF-α | Liver |
|  |  | 263.76 | 23.69 | 5 | 191.03 | 23.35 | 5 |  |  |  | IL-10 | Liver |
|  |  | 73.25 | 13.10 | 5 | 89.35 | 13.57 | 5 |  |  |  | IL-6 | Liver |
|  |  | 50.73 | 8.45 | 5 | 40.65 | 6.57 | 5 |  |  |  | SOD | Liver |
|  |  | 12.76 | 0.73 | 5 | 14.59 | 1.09 | 5 |  |  |  | MDA | Liver |
|  |  | 20.89 | 2.61 | 5 | 25.99 | 2.07 | 5 |  |  |  | Endotoxin | Serum |

**References**

[1] A. Parker, S. Romano, R. Ansorge, A. Aboelnour, G. Le Gall, G.M. Savva, M.G. Pontifex, A. Telatin, D. Baker, E. Jones, D. Vauzour, S. Rudder, L.A. Blackshaw, G. Jeffery, S.R. Carding, Fecal microbiota transfer between young and aged mice reverses hallmarks of the aging gut, eye, and brain, Microbiome 10(1) (2022) 68.

[2] A. Brandt, A. Baumann, A. Hernandez-Arriaga, F. Jung, A. Nier, R. Staltner, D. Rajcic, C. Schmeer, O.W. Witte, B. Wessner, B. Franzke, K.H. Wagner, A. Camarinha-Silva, I. Bergheim, Impairments of intestinal arginine and NO metabolisms trigger aging-associated intestinal barrier dysfunction and 'inflammaging', Redox Biol 58 (2022) 102528.

[3] D. Wang, H. Wang, Y. Li, J. Lu, X. Tang, D. Yang, M. Wang, D. Zhao, F. Liu, S. Zhang, L. Sun, Alistipes senegalensis is Critically Involved in Gut Barrier Repair Mediated by Panax Ginseng Neutral Polysaccharides in Aged Mice, Adv Sci (Weinh) 12(36) (2025) e16427.

[4] S.P. Mishra, S. Jain, B. Wang, S. Wang, B.C. Miller, J.Y. Lee, C.V. Borlongan, L. Jiang, J. Pollak, S. Taraphder, B.T. Layden, S.G. Rane, H. Yadav, Abnormalities in microbiota/butyrate/FFAR3 signaling in aging gut impair brain function, JCI Insight 9(3) (2024).

[5] K.A. Kim, J.J. Jeong, S.Y. Yoo, D.H. Kim, Gut microbiota lipopolysaccharide accelerates inflamm-aging in mice, BMC Microbiol 16 (2016) 9.

[6] L. Wang, Z. Deng, Y. Li, Y. Wu, R. Yao, Y. Cao, M. Wang, F. Zhou, H. Zhu, H. Kang, Ameliorative effects of mesenchymal stromal cells on senescence associated phenotypes in naturally aged rats, J Transl Med 22(1) (2024) 722.

[7] S.E. Webster, D. Vos, T.L. Rothstein, N.E. Holodick, Modulation of microbiome diversity and cytokine expression is influenced in a sex-dependent manner during aging, Front Microbiomes 1 (2022).

[8] P.E. Gamez-Macias, E. Felix-Soriano, M. Samblas, N. Sainz, M.J. Moreno-Aliaga, P. Gonzalez-Muniesa, Intestinal Permeability, Gut Inflammation, and Gut Immune System Response Are Linked to Aging-Related Changes in Gut Microbiota Composition: A Study in Female Mice, J Gerontol A Biol Sci Med Sci 79(4) (2024).

[9] S. Chen, C. Wang, X. Zou, H. Li, G. Yang, X. Su, Z. Mo, Multi-omics insights implicate the remodeling of the intestinal structure and microbiome in aging, Front Genet 15 (2024) 1450064.

[10] Y. Li, L. Ning, Y. Yin, R. Wang, Z. Zhang, L. Hao, B. Wang, X. Zhao, X. Yang, L. Yin, S. Wu, D. Guo, C. Zhang, Age-related shifts in gut microbiota contribute to cognitive decline in aged rats, Aging (Albany NY) 12(9) (2020) 7801-7817.

[11] Y. Morita, K. Jounai, A. Sakamoto, Y. Tomita, Y. Sugihara, H. Suzuki, K. Ohshio, M. Otake, D. Fujiwara, O. Kanauchi, M. Maruyama, Long-term intake of Lactobacillus paracasei KW3110 prevents age-related chronic inflammation and retinal cell loss in physiologically aged mice, Aging (Albany NY) 10(10) (2018) 2723-2740.

[12] R. Vemuri, C. Sherrill, M.A. Davis, K. Kavanagh, Age-Related Colonic Mucosal Microbiome Community Shifts in Monkeys, J Gerontol A Biol Sci Med Sci 76(11) (2021) 1906-1914.

[13] M.N. Conley, C.P. Wong, K.M. Duyck, N. Hord, E. Ho, T.J. Sharpton, Aging and serum MCP-1 are associated with gut microbiome composition in a murine model, PeerJ 4 (2016) e1854.

[14] Y. Jing, Q. Wang, F. Bai, Z. Li, Y. Li, W. Liu, Y. Yan, S. Zhang, C. Gao, Y. Yu, Role of microbiota-gut-brain axis in natural aging-related alterations in behavior, Front Neurosci 18 (2024) 1362239.

[15] V.E. Brunt, T.J. LaRocca, A.E. Bazzoni, Z.J. Sapinsley, J. Miyamoto-Ditmon, R.A. Gioscia-Ryan, A.P. Neilson, C.D. Link, D.R. Seals, The gut microbiome-derived metabolite trimethylamine N-oxide modulates neuroinflammation and cognitive function with aging, Geroscience 43(1) (2021) 377-394.

[16] R. Shi, J. Ye, H. Fan, X. Hu, X. Wu, D. Wang, B. Zhao, X. Dai, X. Liu, Lactobacillus plantarum LLY-606 Supplementation Ameliorates the Cognitive Impairment of Natural Aging in Mice: The Potential Role of Gut Microbiota Homeostasis, J Agric Food Chem 72(8) (2024) 4049-4062.

[17] J. Ni, F. Wang, Y. Wu, J. Shen, Z. Zhou, L. Xu, Z. Lou, Y. Shen, B. Qiu, Q. Xiang, P. Yin, Y. Chen, L. Li, B. adolescentis alleviates inflammation and suppresses PGE2 through remodling gut microbiota to attenuate pulmonary fibrosis in aging mice, NPJ Sci Food 9(1) (2025) 251.

[18] N.A. Crossland, S. Beck, W.Y. Tan, M. Lo, J.B. Mason, C. Zhang, W. Guo, J.W. Crott, Fecal microbiota transplanted from old mice promotes more colonic inflammation, proliferation, and tumor formation in azoxymethane-treated A/J mice than microbiota originating from young mice, Gut Microbes 15(2) (2023) 2288187.

[19] X. Zeng, X. Li, X. Li, C. Wei, C. Shi, K. Hu, D. Kong, Q. Luo, Y. Xu, W. Shan, M. Zhang, J. Shi, J. Feng, Y. Han, H. Huang, P. Qian, Fecal microbiota transplantation from young mice rejuvenates aged hematopoietic stem cells by suppressing inflammation, Blood 141(14) (2023) 1691-1707.

[20] C.K. Cheng, J. Gao, L. Kang, Y. Huang, Fecal Microbiota Transfer from Young Mice Reverts Vascular Aging Hallmarks and Metabolic Impairments in Aged Mice, Aging Dis 16(3) (2024) 1576-1585.

[21] X. Chen, D. Zhu, R. Ge, Z. Bao, Fecal transplantation of young mouse donors effectively improves enterotoxicity in elderly recipients exposed to triphenyltin, Ecotoxicol Environ Saf 273 (2024) 116140.

[22] J. Lee, V.R. Venna, D.J. Durgan, H. Shi, J. Hudobenko, N. Putluri, J. Petrosino, L.D. McCullough, R.M. Bryan, Young versus aged microbiota transplants to germ-free mice: increased short-chain fatty acids and improved cognitive performance, Gut Microbes 12(1) (2020) 1-14.

[23] X. Yang, D. Yu, L. Xue, H. Li, J. Du, Probiotics modulate the microbiota-gut-brain axis and improve memory deficits in aged SAMP8 mice, Acta Pharm Sin B 10(3) (2020) 475-487.

[24] I. Yusufu, K. Ding, K. Smith, U.D. Wankhade, B. Sahay, G.T. Patterson, R. Pacholczyk, S. Adusumilli, M.W. Hamrick, W.D. Hill, C.M. Isales, S. Fulzele, A Tryptophan-Deficient Diet Induces Gut Microbiota Dysbiosis and Increases Systemic Inflammation in Aged Mice, Int J Mol Sci 22(9) (2021).

[25] R.D. Li, W.X. Zheng, Q.R. Zhang, Y. Song, Y.T. Liao, F.C. Shi, X.H. Wei, F. Zhou, X.H. Zheng, K.Y. Tan, Q.Y. Li, Longevity-Associated Core Gut Microbiota Mining and Effect of Mediated Probiotic Combinations on Aging Mice: Case Study of a Long-Lived Population in Guangxi, China, Nutrients 15(7) (2023).

[26] K. Wuttisa, P. Sookpotarom, B. Poopan, C. Chantarangkul, P. Jamjuree, J. Namkaew, T. Jaroonwitchawan, M. Taweechotipatr, The potential of novel gut microbiota supplement in mitigating gut inflammation, alleviating oxidative stress linked to aging, and improving cognitive function in aged mice, BMC Complement Med Ther 25(1) (2025) 137.

[27] Y. Cai, Y. Dong, M. Han, M. Jin, H. Liu, Z. Gai, K. Zou, Lacticaseibacillus paracasei LC86 mitigates age-related muscle wasting and cognitive impairment in SAMP8 mice through gut microbiota modulation and the regulation of serum inflammatory factors, Front Nutr 11 (2024) 1390433.

[28] S.Y. Wang, W.C. Yen, Y.P. Chen, J.S. Shiu, M.J. Chen, Developing a Novel Fermented Milk with Anti-Aging and Anti-Oxidative Properties Using Lactobacillus kefiranofaciens HL1 and Lactococcus lactis APL015, Nutrients 17(15) (2025).

[29] J.J. Jeong, K.A. Kim, S.E. Jang, J.Y. Woo, M.J. Han, D.H. Kim, Orally administrated Lactobacillus pentosus var. plantarum C29 ameliorates age-dependent colitis by inhibiting the nuclear factor-kappa B signaling pathway via the regulation of lipopolysaccharide production by gut microbiota, PLoS One 10(2) (2015) e0116533.

[30] M. Ren, H. Li, Z. Fu, Q. Li, Centenarian-Sourced Lactobacillus casei Combined with Dietary Fiber Complex Ameliorates Brain and Gut Function in Aged Mice, Nutrients 14(2) (2022).

[31] Q. Zeng, Z. Qi, X. He, C. Luo, J. Wen, J. Wei, F. Yue, X. Zhao, H. Wei, T. Chen, Bifidobacterium pseudocatenulatum NCU-08 ameliorated senescence via modulation of the AMPK/Sirt1 signaling pathway and gut microbiota in mice, Food Funct 15(8) (2024) 4095-4108.

[32] X. Liu, K. Guan, C. Liu, Y. Sun, Y. Ma, K. Mao, Q. Li, R. Wang, W. Lu, Qula-derived Limosilactobacillus fermentum TD-3 and Lactococcus lactis MQ1-1 alleviate aging-related intestinal barrier dysfunction via microbiota-short-chain fatty acid-AMPK/MLCK-tight junction axis, J Dairy Sci 108(12) (2025) 12949-12969.

[33] P. Vitheejongjaroen, A. Kasorn, N. Puttarat, F. Loison, M. Taweechotipatr, Bifidobacterium animalis MSMC83 Improves Oxidative Stress and Gut Microbiota in D-Galactose-Induced Rats, Antioxidants (Basel) 11(11) (2022).

[34] H. Han, J. Tao, X. Bai, Y. Jing, Z. Zhai, J. Luo, W. Zhang, D. Gan, Y. Hao, Lychee Fermented by Mixed Probiotic Strains Alleviates D-Galactose-Induced Skeletal Muscle and Intestinal Aging in Mice, Foods 14(21) (2025).

[35] Y. Zeng, J. Wang, L. Zhang, H. Xiao, L. Zhu, X. Wang, W. Gu, M. Xu, Y. Han, S. Wang, Bifidobacterium breve NKU BB 1–13 alleviates age-related cardiac dysfunction by improving colon aging and modulating gut microbiota to influence blood metabolites, Journal of Functional Foods 135 (2025).

[36] J. Fang, Y. Dai, J. Chen, H. Zhang, H. Li, W. Chen, Lactiplantibacillus plantarum CCFM8610 mitigates oxidative stress-related intestinal aging through its metabolite indole-3-lactic acid, Food Bioscience 63 (2025).

[37] H. Nie, X. Wang, Y. Luo, F. Kong, G. Mu, X. Wu, Mechanism Explanation on Improved Cognitive Ability of D-Gal Inducing Aged Mice Model by Lactiplantibacillus plantarum MWFLp-182 via the Microbiota-Gut-Brain Axis, J Agric Food Chem 72(17) (2024) 9795-9806.

[38] F. Chen, J. Pan, L. Yu, S. Wang, C. Zhang, J. Zhao, A. Narbad, Q. Zhai, F. Tian, Lactiplantibacillus plantarum CCFM8661 alleviates D-galactose-induced brain aging in mice by the regulation of the gut microbiota, Food Funct 14(22) (2023) 10135-10150.

[39] L.H. Chen, M.F. Wang, C.C. Chang, S.Y. Huang, C.H. Pan, Y.T. Yeh, C.H. Huang, C.H. Chan, H.Y. Huang, Lacticaseibacillus paracasei PS23 Effectively Modulates Gut Microbiota Composition and Improves Gastrointestinal Function in Aged SAMP8 Mice, Nutrients 13(4) (2021).

[40] L. Wu, H. He, T. Liang, G. Du, L. Li, H. Zhong, Y. Li, J. Zhang, N. Chen, T. Jiang, J. Yang, J. Wang, S. Feng, S. Lu, H. Zhao, Q. Gu, H. Gao, G. Li, W. Xie, L. Wu, Q. Wu, X. Xie, X. He, Mesaconic acid as a key metabolite with anti-inflammatory and anti-aging properties produced by Lactobacillus plantarum 124 from centenarian gut microbiota, NPJ Biofilms Microbiomes 11(1) (2025) 165.
