# Supplementary material for: A Multi‐Organ Atlas Links Gut Microbial Metabolites to Systemic Redox Changes in Aging Mice
Source: Aging Cell. 2026 Mar 9;25(3):e70433. doi: 10.1111/acel.70433 (PMC12968584; doi:10.1111/acel.70433)
Supplement: Supplementary file 1 — Data S1: acel70433‐sup‐0001‐Supinfo.zip. [file ACEL-25-e70433-s001.zip › acel70433-sup-0001-Supinfo/Supplementary Figures.zip!/S1 feces.pdf]

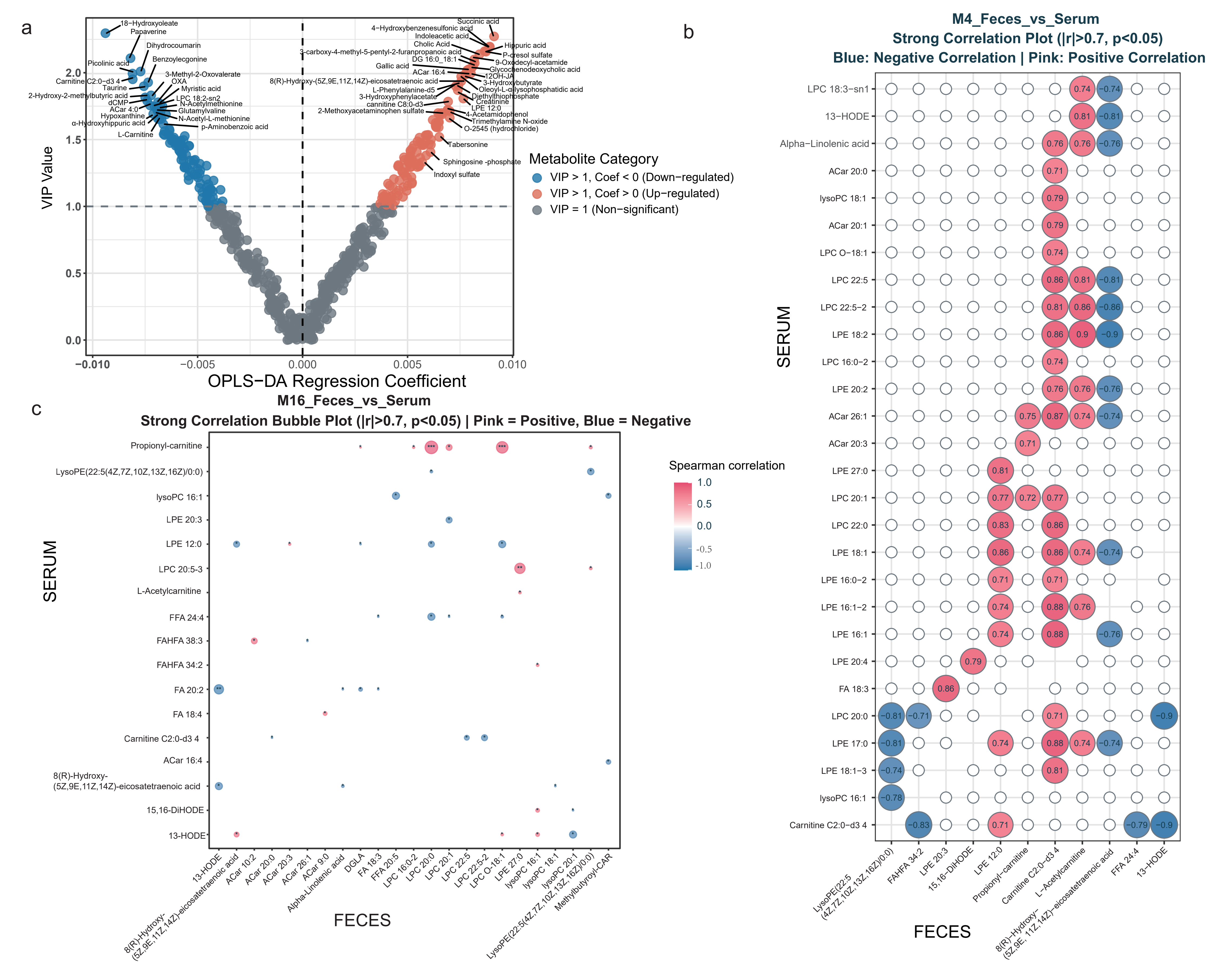
a
VIP Value
OPLS−DA Regression Coefficient
−0.010
−0.005
0.000
0.005
0.010
0.0
0.5
1.0
1.5
2.0
18−Hydroxyoleate
Papaverine
Dihydrocoumarin
Picolinic acid
Benzoylecgonine
Carnitine C2:0-d3 4
3-Methyl-2-Oxovalerate
OXA
Taurine
Myristic acid
2-Hydroxy-2-methylbutyric acid
LPC 18:2-sn2
dCMP
N-Acetylmethionine
ACar 4:0
Glutamylvaline
Hypoxanthine
N-Acetyl-L-methionine
α-Hydroxyhippuric acid
p-Aminobenzoic acid
L-Carnitine
Succinic acid
4−Hydroxybenzenesulfonic acid
Indoleacetic acid
Hippuric acid
Cholic Acid
3-carboxy-4-methyl-5-pentyl-2-furanpropanoic acid
P-cresol sulfate
DG 16:0_18:1
9-Oxodecyl-acetamide
Gallic acid
Glycochenodeoxycholic acid
ACar 16:4
12OH-JA
8(R)-Hydroxy-(5Z,9E,11Z,14Z)-eicosatetraenoic acid
3-Hydroxybutyrate
L-Phenylalanine-d5
Oleoyl-L-α-lysophosphatidic acid
3-Hydroxyphenylacetate
Diethylthiophosphate
cannitine C8:0-d3
Creatinine
LPE 12:0
2-Methoxyacetaminophen sulfate
4-Acetamidophenol
Trimethylamine N-oxide
O-2545 (hydrochloride)
Tabersonine
Sphingosine -phosphate
Indoxyl sulfate
Metabolite Category
VIP > 1, Coef < 0 (Down−regulated)
VIP > 1, Coef > 0 (Up−regulated)
VIP = 1 (Non−significant)
b
M4_Feces_vs_Serum
Strong Correlation Plot (|r|>0.7, p<0.05)
Blue: Negative Correlation | Pink: Positive Correlation
SERUM
FECES
LPC 18:3−sn1
13−HODE
Alpha−Linolenic acid
ACar 20:0
lysoPC 18:1
ACar 20:1
LPC O−18:1
LPC 22:5
LPC 22:5−2
LPE 18:2
LPC 16:0−2
LPE 20:2
ACar 26:1
ACar 20:3
LPE 27:0
LPC 20:1
LPC 22:0
LPE 18:1
LPE 16:0−2
LPE 16:1−2
LPE 16:1
LPE 20:4
FA 18:3
LPC 20:0
LPE 17:0
LPE 18:1−3
lysoPC 16:1
Carnitine C2:0−d3 4
LysoPE(22:5 (4Z,7Z,10Z,13Z,16Z)/0:0)
FAHFA 34:2
LPE 20:3
15,16−DiHODE
LPE 12:0
Propionyl−carnitine
Carnitine C2:0−d3 4
L−Acetylcarnitine
8(R)−Hydroxy− (5Z,9E, 11Z,14Z)−eicosatetraenoic acid
FFA 24:4
13−HODE
c
M16_Feces_vs_Serum
Strong Correlation Bubble Plot (|r|>0.7, p<0.05) | Pink = Positive, Blue = Negative
SERUM
FECES
Propionyl-carnitine
LysoPE(22:5(4Z,7Z,10Z,13Z,16Z)/0:0)
lysoPC 16:1
LPE 20:3
LPE 12:0
LPC 20:5-3
L-Acetylcarnitine
FFA 24:4
FAHFA 38:3
FAHFA 34:2
FA 20:2
FA 18:4
Carnitine C2:0-d3 4
ACar 16:4
8(R)-Hydroxy- (5Z,9E,11Z,14Z)-eicosatetraenoic acid
15,16-DiHODE
13-HODE
13-HODE
8(R)-Hydroxy- (5Z,9E,11Z,14Z)-eicosatetraenoic acid
ACar 10:2
ACar 20:0
ACar 20:3
ACar 26:1
ACar 9:0
Alpha-Linolenic acid
DGLA
FA 18:3
FFA 20:5
LPC 16:0-2
LPC 20:0
LPC 20:1
LPC 22:5
LPC 22:5-2
LPC O-18:1
LPE 27:0
lysoPC 16:1
lysoPC 18:1
lysoPC 20:1
LysoPE(22:5(4Z,7Z,10Z,13Z,16Z)/0:0)
Methylbutyroyl-CAR
Spearman correlation
1.0
0.5
0.0
-0.5
-1.0
