# Supplementary material for: A Multi‐Organ Atlas Links Gut Microbial Metabolites to Systemic Redox Changes in Aging Mice
Source: Aging Cell. 2026 Mar 9;25(3):e70433. doi: 10.1111/acel.70433 (PMC12968584; doi:10.1111/acel.70433)
Supplement: Supplementary file 1 — Data S1: acel70433‐sup‐0001‐Supinfo.zip. [file ACEL-25-e70433-s001.zip › acel70433-sup-0001-Supinfo/Supplementary Figures.zip!/S2 serum.pdf]

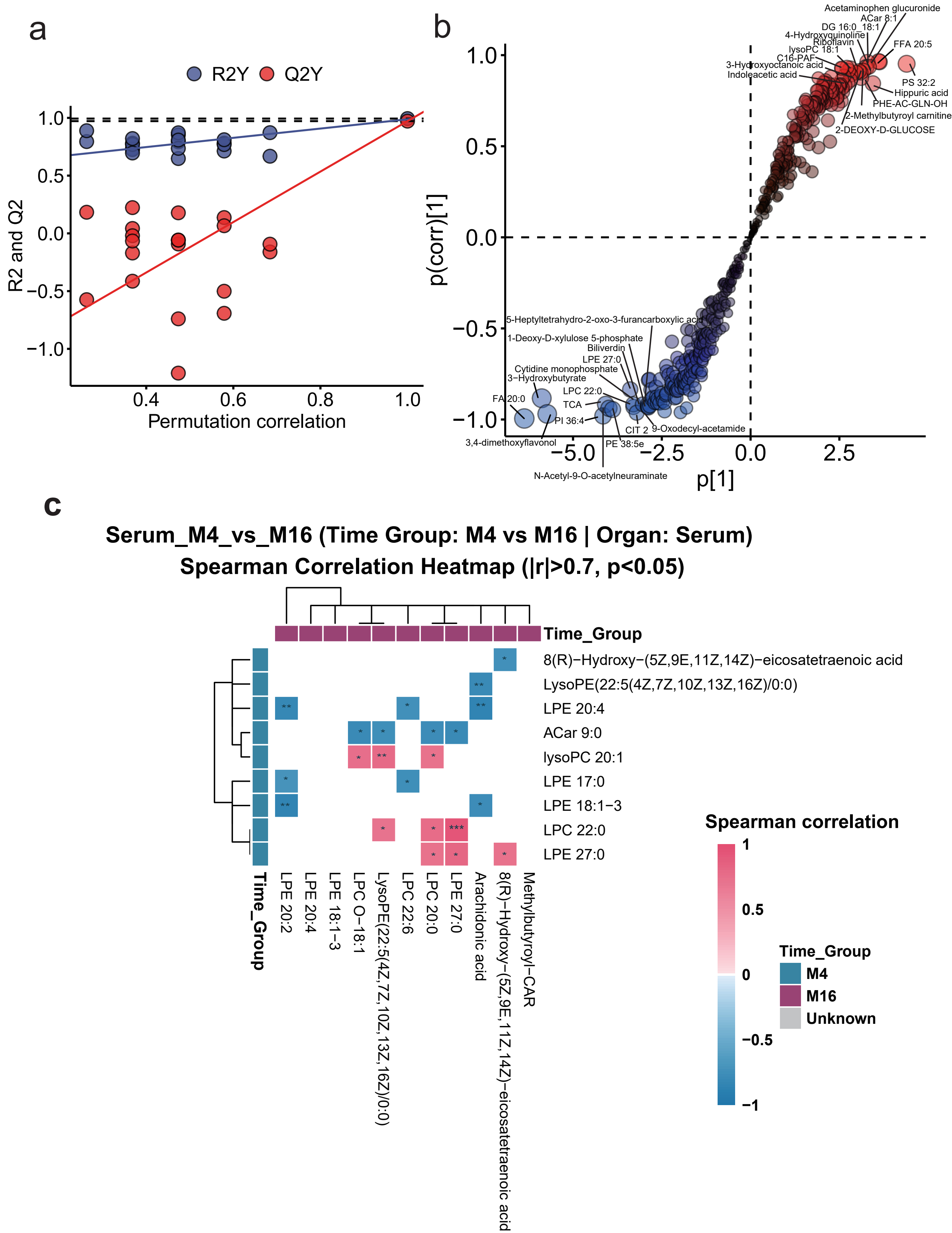
a
R2Y
Q2Y
R2 and Q2
Permutation correlation
1.0
0.5
0.0
−0.5
−1.0
0.4
0.6
0.8
1.0
b
p(corr)[1]
p[1]
−5.0
−2.5
0.0
2.5
Acetaminophen glucuronide
ACar 8:1
DG 16:0_18:1
4-Hydroxyquinoline
Riboflavin
lysoPC 18:1
C16-PAF
3-Hydroxyoctanoic acid
Indoleacetic acid
FFA 20:5
PS 32:2
Hippuric acid
PHE-AC-GLN-OH
2-Methylbutyroyl carnitine
2-DEOXY-D-GLUCOSE
5-Heptyltetrahydro-2-oxo-3-furancarboxylic acid
1-Deoxy-D-xylulose 5-phosphate
Biliverdin
LPE 27:0
Cytidine monophosphate
3−Hydroxybutyrate
LPC 22:0
FA 20:0
TCA
PI 36:4
CIT 2
9-Oxodecyl-acetamide
PE 38:5e
3,4-dimethoxyflavonol
N-Acetyl-9-O-acetylneuraminate
c
Serum_M4_vs_M16 (Time Group: M4 vs M16 | Organ: Serum)
Spearman Correlation Heatmap (|r|>0.7, p<0.05)
Time_Group
8(R)−Hydroxy−(5Z,9E,11Z,14Z)−eicosatetraenoic acid
LysoPE(22:5(4Z,7Z,10Z,13Z,16Z)/0:0)
LPE 20:4
ACar 9:0
lysoPC 20:1
LPE 17:0
LPE 18:1−3
LPC 22:0
LPE 27:0
Time_Group
LPE 20:2
LPE 20:4
LPE 18:1−3
LPC O−18:1
LysoPE(22:5(4Z,7Z,10Z,13Z,16Z)/0:0)
LPC 22:6
LPC 20:0
LPE 27:0
Arachidonic acid
8(R)−Hydroxy−(5Z,9E,11Z,14Z)−eicosatetraenoic acid
Methylbutyroyl−CAR
Spearman correlation
1
0.5
0
−0.5
−1
Time_Group
M4
M16
Unknown
