# Supplementary material for: A Multi‐Organ Atlas Links Gut Microbial Metabolites to Systemic Redox Changes in Aging Mice
Source: Aging Cell. 2026 Mar 9;25(3):e70433. doi: 10.1111/acel.70433 (PMC12968584; doi:10.1111/acel.70433)
Supplement: Supplementary file 1 — Data S1: acel70433‐sup‐0001‐Supinfo.zip. [file ACEL-25-e70433-s001.zip › acel70433-sup-0001-Supinfo/Supplementary Figures.zip!/S3 liver.pdf]

a

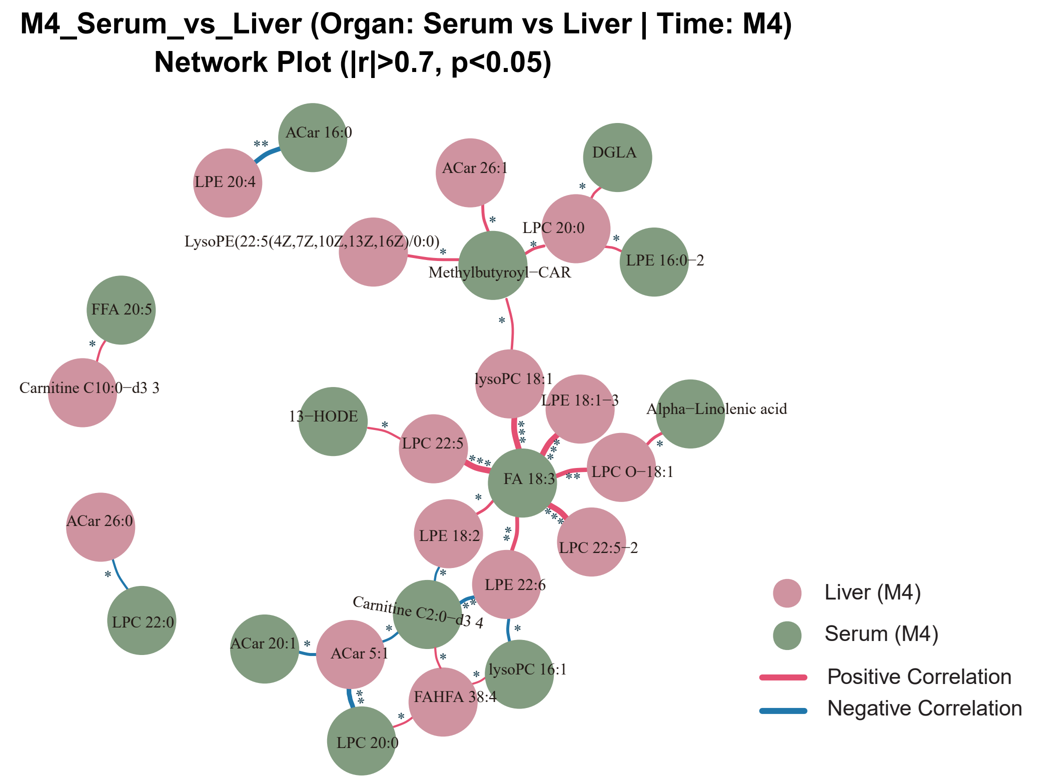
M4_Serum_vs_Liver (Organ: Serum vs Liver | Time: M4)
Network Plot (|r|>0.7, p<0.05)
ACar 16:0
LPE 20:4
ACar 26:1
DGLA
LPC 20:0
LysoPE(22:5(4Z,7Z,10Z,13Z,16Z)/0:0)
Methylbutyroyl−CAR
LPE 16:0−2
FFA 20:5
Carnitine C10:0−d3 3
lysoPC 18:1
LPE 18:1−3
Alpha−Linolenic acid
13−HODE
LPC 22:5
FA 18:3
LPC O−18:1
ACar 26:0
LPE 18:2
LPC 22:5−2
LPE 22:6
Carnitine C2:0−d3 4
LPC 22:0
ACar 20:1
ACar 5:1
lysoPC 16:1
FAHFA 38:4
LPC 20:0
Liver (M4)
Serum (M4)
Positive Correlation
Negative Correlation

b

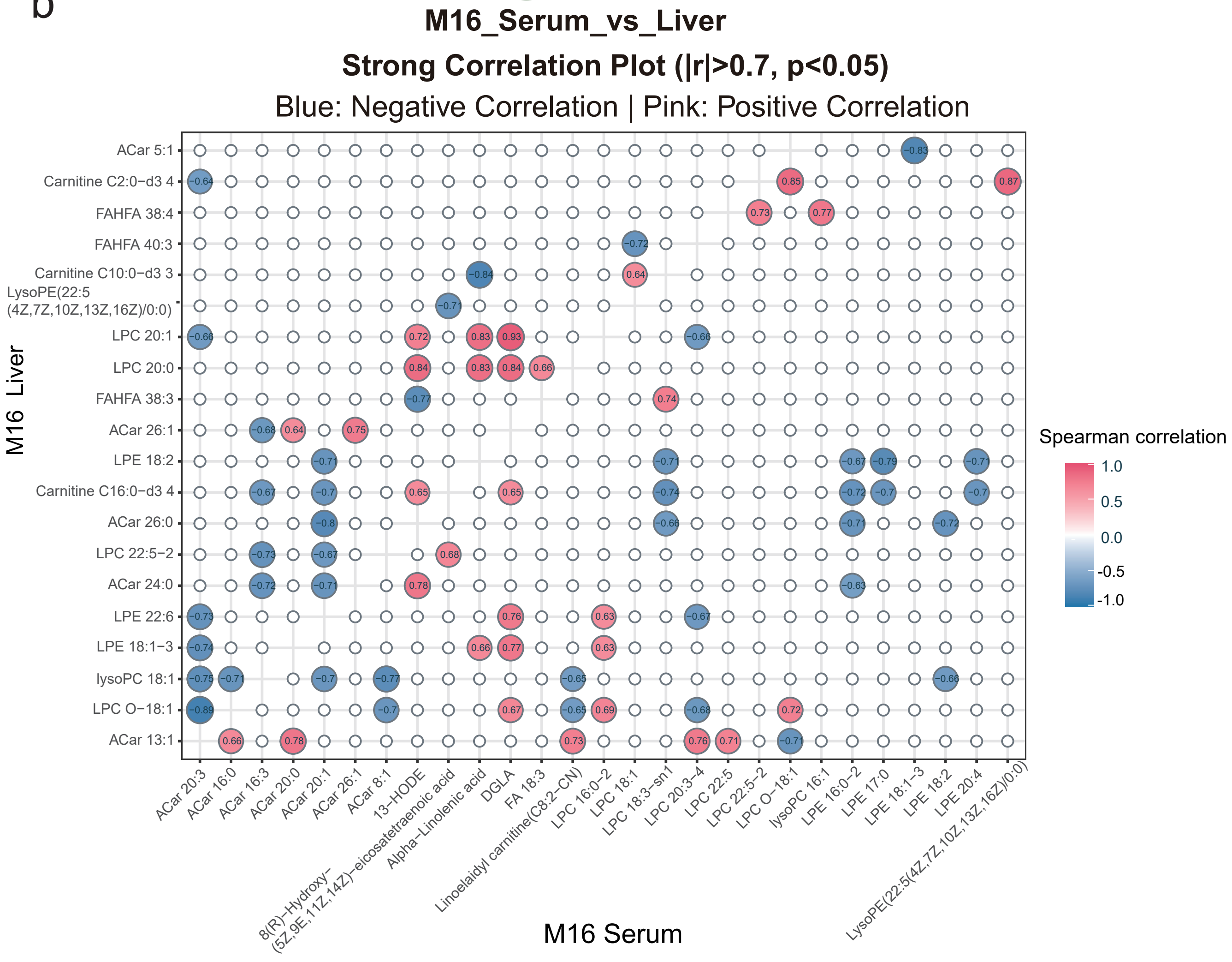
M16_Serum_vs_Liver
Strong Correlation Plot (|r|>0.7, p<0.05)
Blue: Negative Correlation | Pink: Positive Correlation
M16 Liver
ACar 5:1
Carnitine C2:0−d3 4
FAHFA 38:4
FAHFA 40:3
Carnitine C10:0−d3 3
LysoPE(22:5 (4Z,7Z,10Z,13Z,16Z)/0:0)
LPC 20:1
LPC 20:0
FAHFA 38:3
ACar 26:1
LPE 18:2
Carnitine C16:0−d3 4
ACar 26:0
LPC 22:5−2
ACar 24:0
LPE 22:6
LPE 18:1−3
lysoPC 18:1
LPC O−18:1
ACar 13:1
ACar 20:3
ACar 16:0
ACar 16:3
ACar 20:0
ACar 20:1
ACar 26:1
ACar 8:1
13−HODE
8(R)−Hydroxy− (5Z,9E,11Z,14Z)−eicosatetraenoic acid
Alpha−Linolenic acid
DGLA
FA 18:3
Linoelaidyl carnitine(C8:2−CN)
LPC 16:0−2
LPC 18:1
LPC 18:3−sn1
LPC 20:3−4
LPC 22:5
LPC 22:5−2
LPC O−18:1
lysoPC 16:1
LPE 16:0−2
LPE 17:0
LPE 18:1−3
LPE 18:2
LPE 20:4
LysoPE(22:5(4Z,7Z,10Z,13Z,16Z)/0:0)
M16 Serum
Spearman correlation
1.0
0.5
0.0
-0.5
-1.0
