# Supplementary material for: A Multi‐Organ Atlas Links Gut Microbial Metabolites to Systemic Redox Changes in Aging Mice
Source: Aging Cell. 2026 Mar 9;25(3):e70433. doi: 10.1111/acel.70433 (PMC12968584; doi:10.1111/acel.70433)
Supplement: Supplementary file 1 — Data S1: acel70433‐sup‐0001‐Supinfo.zip. [file ACEL-25-e70433-s001.zip › acel70433-sup-0001-Supinfo/Supplementary Figures.zip!/S4 lung.pdf]

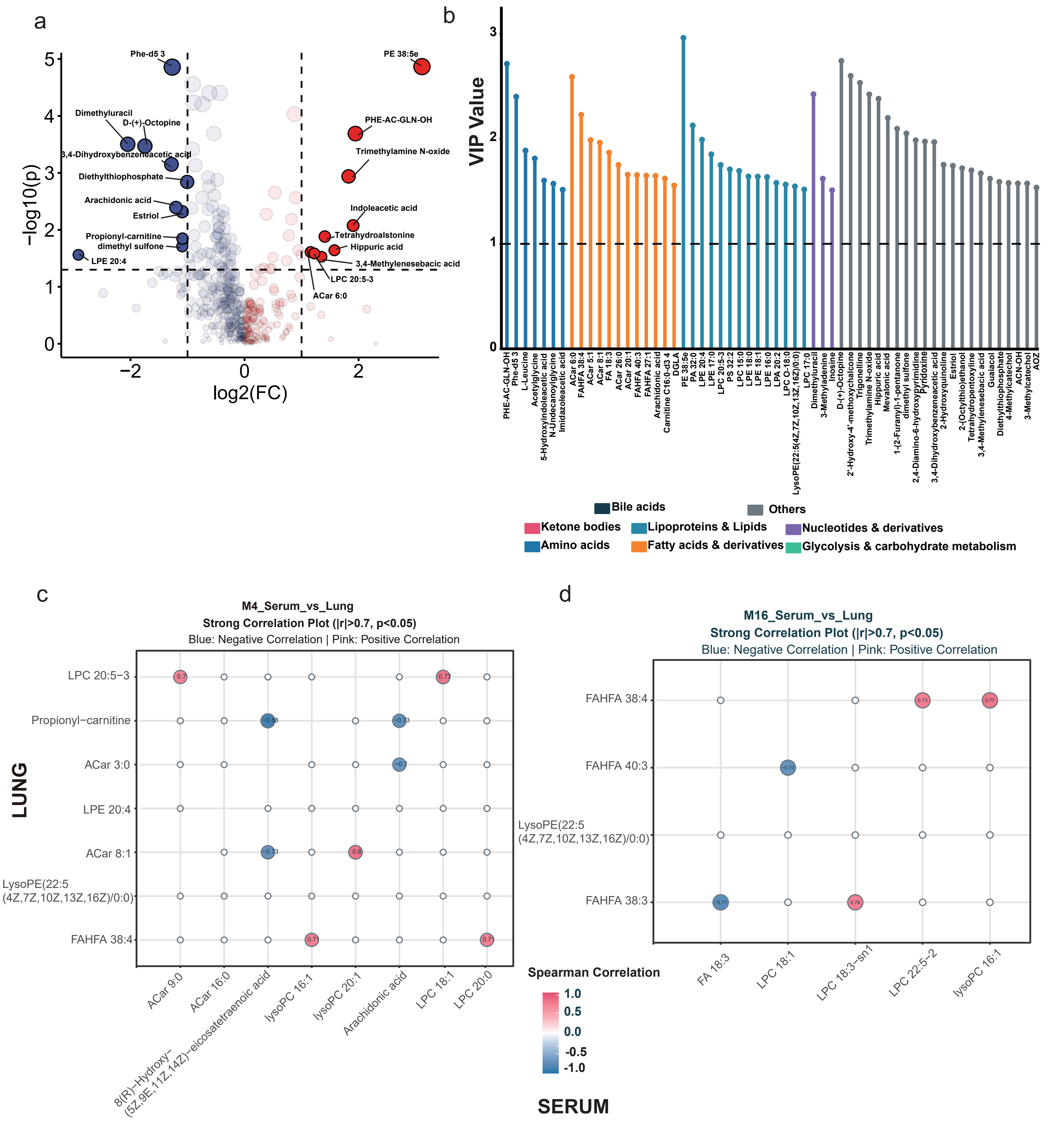
a
b
c
d
M4_Serum_vs_Lung
Strong Correlation Plot (|r|>0.7, p<0.05)
Blue: Negative Correlation | Pink: Positive Correlation
M16_Serum_vs_Lung
Strong Correlation Plot (|r|>0.7, p<0.05)
Blue: Negative Correlation | Pink: Positive Correlation
Spearman Correlation
LUNG
SERUM
VIP Value
log2(FC)
−log10(p)
Bile acids
Others
Ketone bodies
Lipoproteins & Lipids
Nucleotides & derivatives
Amino acids
Fatty acids & derivatives
Glycolysis & carbohydrate metabolism
