# Supplementary material for: A Multi‐Organ Atlas Links Gut Microbial Metabolites to Systemic Redox Changes in Aging Mice
Source: Aging Cell. 2026 Mar 9;25(3):e70433. doi: 10.1111/acel.70433 (PMC12968584; doi:10.1111/acel.70433)
Supplement: Supplementary file 1 — Data S1: acel70433‐sup‐0001‐Supinfo.zip. [file ACEL-25-e70433-s001.zip › acel70433-sup-0001-Supinfo/Supplementary Figures.zip!/S5 cortex.pdf]

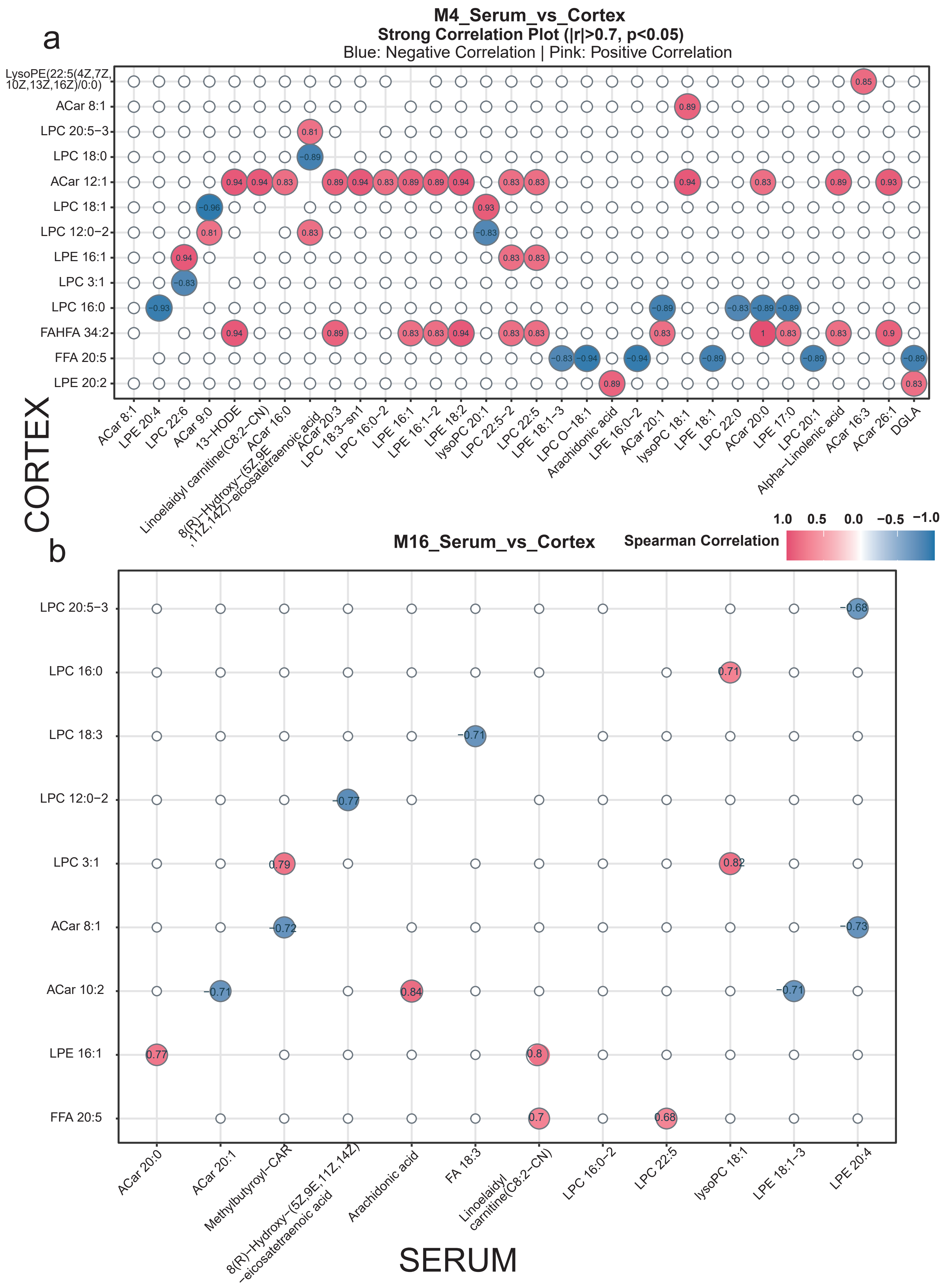

a
M4_Serum_vs_Cortex
Strong Correlation Plot (|r|>0.7, p<0.05)
Blue: Negative Correlation | Pink: Positive Correlation
LysoPE(22:5(4Z,7Z,10Z,13Z,16Z)/0:0)
ACar 8:1
LPC 20:5−3
LPC 18:0
ACar 12:1
LPC 18:1
LPC 12:0−2
LPE 16:1
LPC 3:1
LPC 16:0
FAHFA 34:2
FFA 20:5
LPE 20:2
ACar 8:1
LPE 20:4
LPC 22:6
ACar 9:0
13−HODE
Linoelaidyl carnitine(C8:2−CN)
ACar 16:0
8(R)−Hydroxy−(5Z,9E,11Z,14Z)−eicosatetraenoic acid
ACar 20:3
LPC 18:3−sn1
LPC 16:0−2
LPE 16:1
LPE 16:1−2
LPE 18:2
lysoPC 20:1
LPC 22:5−2
LPC 22:5
LPE 18:1−3
LPC O−18:1
Arachidonic acid
LPE 16:0−2
ACar 20:1
lysoPC 18:1
LPE 18:1
LPC 22:0
ACar 20:0
LPE 17:0
LPC 20:1
Alpha−Linolenic acid
ACar 16:3
ACar 26:1
DGLA
CORTEX
b
M16_Serum_vs_Cortex
Spearman Correlation
1.0 0.5 0.0 −0.5 −1.0
LPC 20:5−3
LPC 16:0
LPC 18:3
LPC 12:0−2
LPC 3:1
ACar 8:1
ACar 10:2
LPE 16:1
FFA 20:5
ACar 20:0
ACar 20:1
Methylbutyroyl−CAR
8(R)−Hydroxy−(5Z,9E,11Z,14Z)−eicosatetraenoic acid
Arachidonic acid
FA 18:3
Linoelaidyl carnitine(C8:2−CN)
LPC 16:0−2
LPC 22:5
lysoPC 18:1
LPE 18:1−3
LPE 20:4
SERUM
